# Multiscale co-simulation design pattern for neuroscience applications

**DOI:** 10.3389/fninf.2024.1156683

**Published:** 2024-02-12

**Authors:** Lionel Kusch, Sandra Diaz-Pier, Wouter Klijn, Kim Sontheimer, Christophe Bernard, Abigail Morrison, Viktor Jirsa

**Affiliations:** ^1^Institut de Neurosciences des Systèmes (INS), UMR1106, Aix-Marseille Université, Marseilles, France; ^2^Simulation and Data Lab Neuroscience, Jülich Supercomputing Centre (JSC), Institute for Advanced Simulation, JARA, Forschungszentrum Jülich GmbH, Jülich, Germany; ^3^Forschungszentrum Jülich GmbH, IAS-6/INM-6, JARA, Jülich, Germany; ^4^Computer Science 3 - Software Engineering, RWTH Aachen University, Aachen, Germany

**Keywords:** co-simulation, multiscale, brain network model, spiking neural network, mouse brain

## Abstract

Integration of information across heterogeneous sources creates added scientific value. Interoperability of data, tools and models is, however, difficult to accomplish across spatial and temporal scales. Here we introduce the toolbox Parallel Co-Simulation, which enables the interoperation of simulators operating at different scales. We provide a software science co-design pattern and illustrate its functioning along a neuroscience example, in which individual regions of interest are simulated on the cellular level allowing us to study detailed mechanisms, while the remaining network is efficiently simulated on the population level. A workflow is illustrated for the use case of The Virtual Brain and NEST, in which the CA1 region of the cellular-level hippocampus of the mouse is embedded into a full brain network involving micro and macro electrode recordings. This new tool allows integrating knowledge across scales in the same simulation framework and validating them against multiscale experiments, thereby largely widening the explanatory power of computational models.

## 1 Introduction

The brain is a complex system that includes billions of cells that interact with each other in a nonlinear manner. As a result, even if we were able to measure what all cells are doing simultaneously, we would not necessarily gain a deeper understanding of how the brain works. It has been previously claimed that emergent properties can be only understood through an integrated approach (Cilliers, 2008), ideally in a common theoretical framework to give meaning to data at all scales (Frégnac, 2021). Such a framework using theoretical models can account for nonlinearities and subsequently explain emergent properties (Pillai and Jirsa, 2017; Jirsa and Sheheitli, 2022). Numerous models have been developed to study the interactions of molecules within cells, cell physiology, the activity of cell populations, full brain dynamics and human behavior (Finkelstein et al., 2004; Huys et al., 2014; Einevoll et al., 2019). It is currently impossible to model the brain with all its cellular and molecular constituents due to limitations in resolution, computational resources, or available data from measurements. As a result, even if a given physio/pathological process can be modeled at the macroscopic scale, the lack of microscopic resolution at the molecular scale prevents obtaining mechanistic insight (Meier-Schellersheim et al., 2009). It is, therefore, important to bridge different scales, which is a challenge not unique to neuroscience. In material science, the study of composite materials requires the description of molecular interactions of individual composites and a global description for the analysis of the subsequent deformation of the composite plate (Schlick et al., 2021). In biology, to understand the effect of drugs on tumor growth, it is necessary to model the tissue of cells around the tumor, the tumor cells, and the subcellular transduction signaling pathways (Rejniak and Anderson, 2011; Rahman et al., 2017). In neuroscience, synaptic plasticity uses mechanisms of spike timing on the millisecond scale but leads to the formation of long-term memory evolving on the scale of minutes, days and weeks (Durstewitz et al., 2011).

Our current study aims to provide a methodology to address the scientific and technical problems of multiscale co-simulation in the brain. The main difficulty of multiscale simulation is to enable the information exchange between models formulated at different scales. Such communication can be interpreted as a coupling across scales. For example, in the case of tumors, the tissue around the tumors is represented by a continuum model (first scale), which interacts with discrete tumor cells (second scale); while continuous signaling pathways are modeled in cells (third scale). At present, it is not possible to create a common coupling function amongst these three scales and each scale can use a dedicated simulator engine for optimizing the simulation. In the case of tumors, a common approach is to use COMSOL Multiphysics (COMSOL, 2019) for the tissue simulation, and Matlab (MATLAB, 2017) for the simulation at cellular and subcellular scales. Because the interaction of simulator engines is not a commonly supported feature, co-simulation of models at different scales and within a common framework is challenging. Existing solutions for co-simulation in physics (Gomes et al., 2018; Fish et al., 2021) or in biology (Hetherington et al., 2007; Matthews and Marshall-Coln, 2021) cannot be easily adapted in neuroscience due to the specificity of simulators and models. There is a large number of scale-specific simulators in neuroscience, e.g., for compartmental neurons: Neuron (Carnevale and Hines, 2006), Arbor (Akar et al., 2019), Genesis (Bower and Beeman, 1998); for point neurons: NEST (Gewaltig and Diesmann, 2007), Brian (Stimberg et al., 2019), ANNarchy (Vitay et al., 2015); for the brain network: The Virtual Brain (TVB) (Sanz Leon et al., 2013), Neurolib (Cakan et al., 2023). Most of these simulators can support multiscale simulation to a limited degree, but they remain specialized and optimized for supporting a specific model type; consequently, the usage of other model types diminishes their optimal performance. The objective of co-simulation is to remove this limitation by exploiting the advantages of each simulator within the same simulation (Goddard et al., 2001; Djurfeldt et al., 2010; Mitchinson et al., 2010; Falotico et al., 2017).

Schirner et al. (2022) provide an overview of software tools available for TVB in the European digital neuroscience infrastructure EBRAINS. Two toolboxes for co-simulation are introduced in EBRAINS, TVB-Multiscale and Parallel Co-Simulation. The former toolbox focuses on rapid development for scientific use cases, whereas the latter focuses on optimisation of co-simulation performance and applies the co-simulation design pattern presented in this study. An illustrative example of co-simulation of multiscale models using TVB Multiscale co-simulation is virtual deep brain stimulation (Meier et al., 2022; Shaheen et al., 2022).

Here we present the methodology of the Parallel Co-Simulation toolbox and illustrate its use along the example of combined microscopic Local Field Potential (LFP) and neuronal firing recordings, and macroscopic electro-COrticoGraphy (ECOG) in mice (Renz et al., 2020). This example aims to demonstrate computational requirements for interpreting recorded multiscale data using multiscale modeling (D'Angelo and Jirsa, 2022). The method is based on a software science co-design pattern (Dudkowski, 2009) that dictates the separation of science and technical attributes, allowing these to be addressed in isolation where possible. This separation is based on transformer modules, which synchronize and connect simulators and include the function for transforming data between scales. A multiscale model is built from experimental data obtained in the mouse brain with ECOG cortical signals and LFP signals in the CA1 region of the hippocampus. We co-simulate the model using the simulators TVB and NEST and demonstrate the performance and limitations of the approach along three concrete examples of multiscale network dynamics. The following sections describe the technical details and the optimisation for co-simulation.

## 2 Results

**Figure 1:**
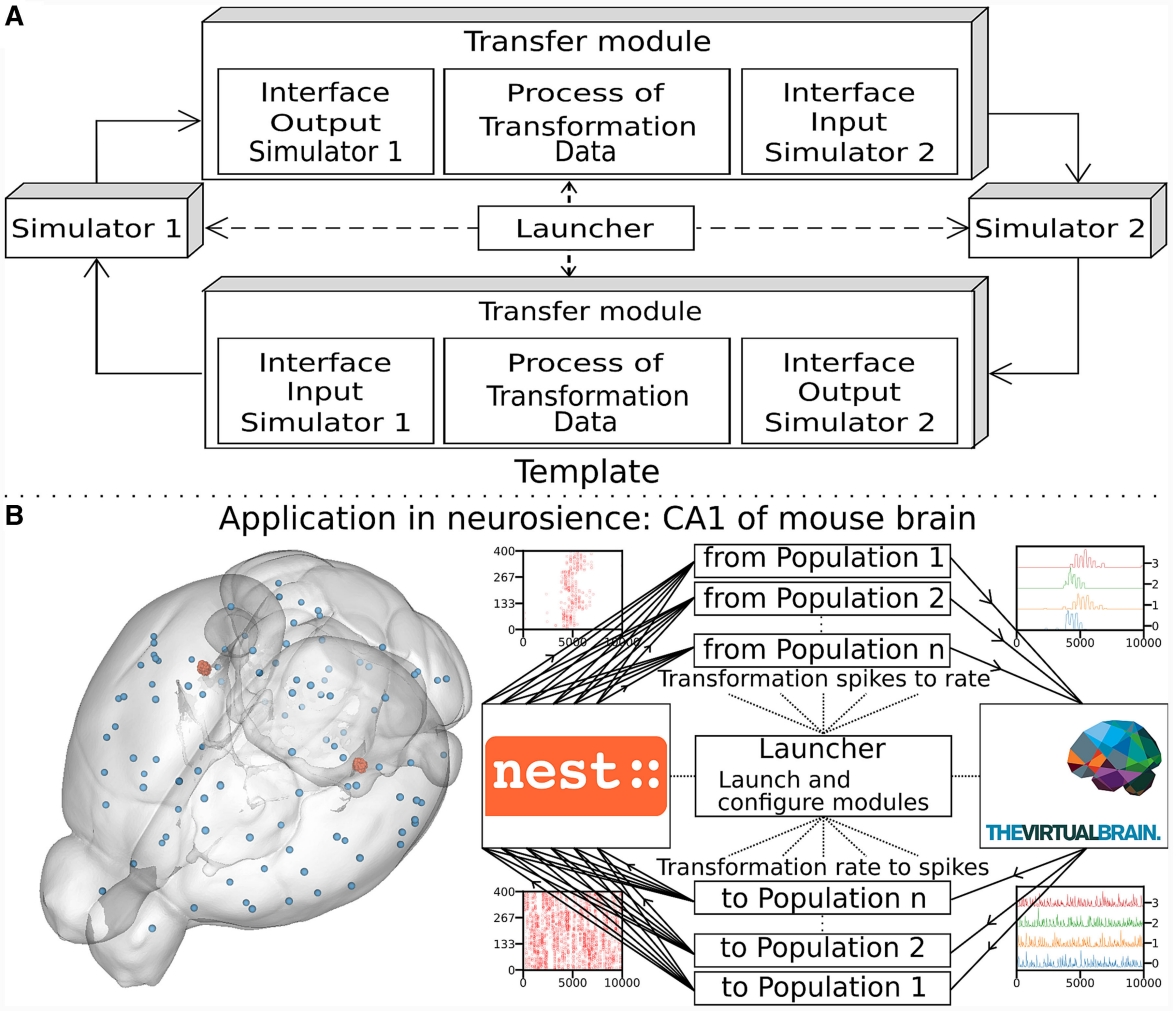
Multiscale co-simulation design pattern and example of an application in neuroscience. **(A)** Multiscale co-simulation design pattern between two simulators using transfer modules to transform and transfer data between scales. **(B)** Application of the co-simulation pattern for a neuroscience use case focusing on the CA1 region of a mouse brain. **Left** shows a rendering of the mouse brain from Allen Institute (Lein et al., 2007). Blue spheres mark the centers of mouse brain regions, and the red spheres are a subset of neurons of the CA1. **Right** illustrates the co-simulation data flow between TVB (Sanz Leon et al., 2013) and NEST (Hahne et al., 2021), showing the different functional modules. The four corners' plots illustrate the data type exchanged in respective information channels. The transfer modules exchange mean firing rate data with TVB (module on the right) and exchange spike times with NEST (module on the left). Each population has a specific module enabling data transfer between populations in different scales.

The multiscale co-simulation software science co-design pattern formalizes the interactions between parallel simulations at different scales. The data transformation among scales is performed during their transfer among simulators. This design pattern comprises five modules (Figure 1A): one launcher, two simulators, and two transfer modules. Each transfer module contains three components: one interface for receiving data, one interface for sending data and a transformation process. The launcher starts and handles the coordination of simulation parameters. The simulators perform scale-specific simulations. The transfer modules transfer the data from one simulator to another. During the transfer, the transformation process transforms the incoming data for the simulator on the receiver side.

This study applies the multiscale co-simulation design pattern to a virtual experiment workflow between the *in-silico* mouse whole-brain dynamics and the *in-silico* micro-scale network dynamics of the hippocampus CA1 region. The recording sites of the virtual CA1 and virtual mouse brain are located at similar positions (Renz et al., 2020) (see Figure 1B). The Virtual Brain (TVB) (Sanz Leon et al., 2013), an open-source platform, has been used to simulate the mouse whole-brain network activity, while NEST (Hahne et al., 2021), another open-source platform, has been employed for the simulation of the CA1 neuronal network dynamics. This specific application illustrates this novel design pattern's technical limitations and demonstrates the potential for a wider range of applications.

### 2.1 Virtual experiment of hippocampal CA1 embedded in a full mouse brain

**Figure 2:**
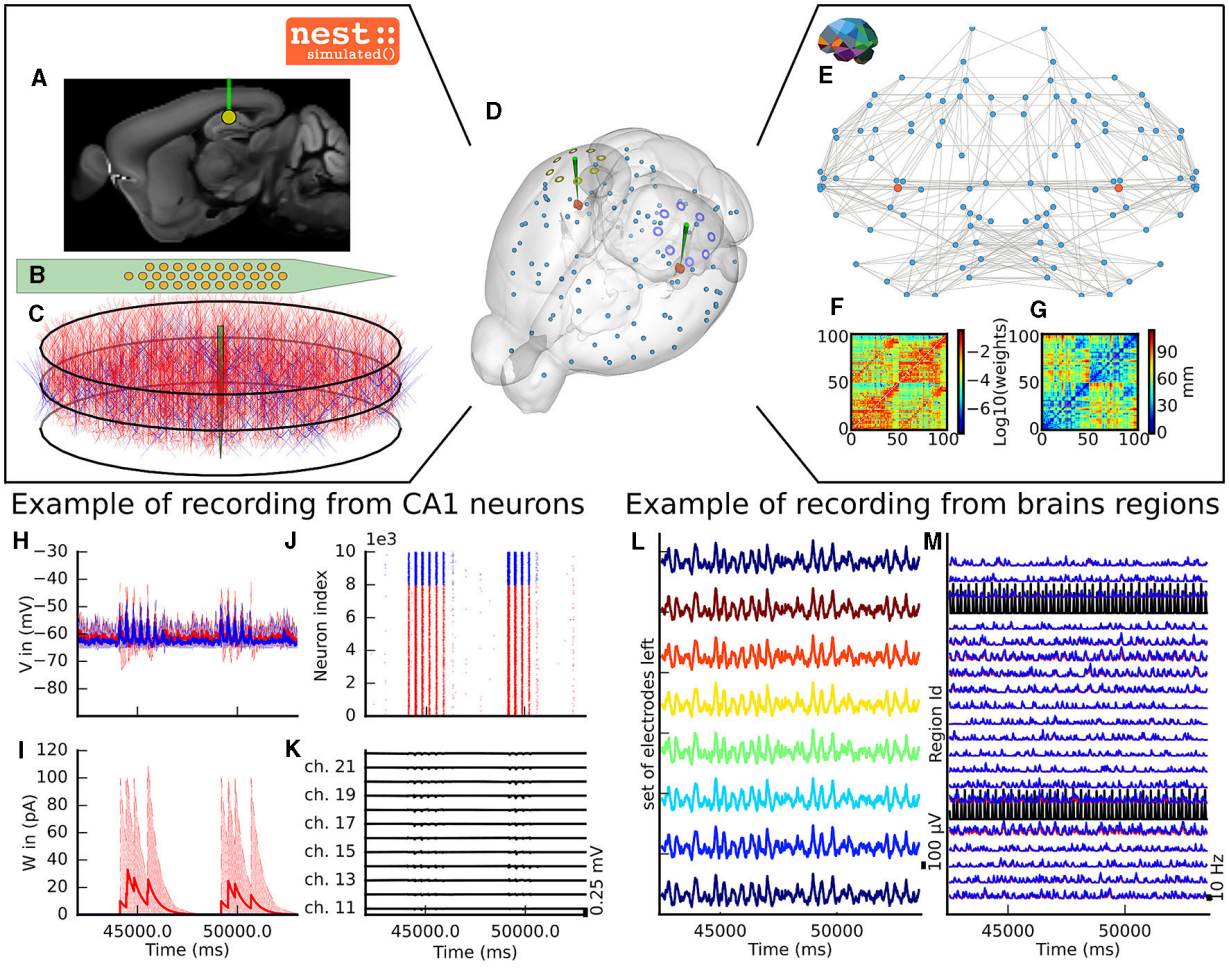
The virtual mouse brain experiment. **(A)** Cross section of the mouse brain with the position of the left implanted electrode. **(B)** Position of the site layout of a polytrode (Neuronexus 32 models from MEAutility library). **(C)** The probe position inside the neural network. The red neurons are pyramidal neurons (Shuman et al., 2020) and the blue neurons are basket cells (Shuman et al., 2020). **(D)** Mouse brain of Allen Institute (Lein et al., 2007) with the position of the two polytrodes and 16 ECOG electrodes. The ECOG electrodes measure the neural field from the surface of the electrode in blue for the left hemisphere and yellow for the right hemisphere. Blue spheres mark the centers of mouse brain regions, and the red spheres are a subset of neurons of the CA1. **(E)** Representation of the connectome of the mouse brain (Oh et al., 2014). The blue dots are brain regions, and the red ones are CA1 regions, whose neurons are simulated with NEST. The gray links highlight the strongest anatomical connections. **(F)** The weights of the anatomical links in graphic F are shown as an adjacency matrix. **(G)** The tract lengths associated with F are shown as an adjacency matrix. The anatomical connections are extracted from tracer data of the Allen Institute (Oh et al., 2014). **(H)** Example of voltage recorded from 10 excitatory and 10 inhibitory neurons. **(I)** Example of adaptation currents recorded from 10 inhibitory and 10 excitatory neurons. **(J)** Example of spike trains recording from the left CA1. **(K)** Example of Local Field Potential recorded from the poly-electrode generated from the spike trains and neuron morphologies. **(L)** Example of recording from the ECOG electrodes of the left hemisphere. **(M)** Example of mean firing rate of excitatory and inhibitory populations for a subset of mouse brain regions.

The virtual experiment of the mouse brain is composed of a brain network model, regional neuronal network models and electrophysiological sensor models. The whole-brain animal model is a network comprised of nodes and edges, where each node contains a neural mass model to simulate the activity of each region and where edges represent the anatomical connections among the regions. The anatomical connections are defined by track lengths and an adjacency matrix representing the coupling strengths of connections between the regions of the network, the “connectome”, which are extracted from tracer data from the Allen Institute (Oh et al., 2014) (Figures 2F, G). The dynamic activity of each brain region is obtained with the neural mass model described by di Volo et al. (2019) (see Section 3). The neuroinformatics platform The Virtual Brain (TVB) (Sanz Leon et al., 2013) performs the animal whole-brain simulation by considering both the chosen neural mass model and specific “connectome”.

The dynamics of the two main brain regions of interest, the left and right hippocampus CA1 (Figure 2), are modeled as a separate neural network composed of point neurons connected with static synapses. Each network comprises one inhibitory, and one excitatory homogeneous population of adaptive exponential integrate and fire neurons (Brette and Gerstner, 2005) (see Section 3). In each microcircuit, the populations of point neurons are taken to be homogeneous; that is, neurons of the same population have the same parameter values. The neuroinformatics platform NEST (Hahne et al., 2021) is able to perform the regional neuronal network simulation using the aforementioned description of the microcircuit of point neurons.

To compare the simulations with empirical data, the virtual experiment contains two models of electrophysiology sensors for probing neural activity. The electrophysiological sensor models are two surface grids of 8-channel electrocorticography arrays and two penetrating multi-electrode arrays of 32 recording sites each. Their positions are illustrated in Figure 2A. Figure 2E shows the position of the polytrodes in the mouse brain, while Figures 2B, D depict the position of the left probes in a cross-section of the left hemisphere and the position of the point of the polytrodes in the population of neurons, respectively. Figure 2C displays the polytrodes with the 32 recording sites. The simulated signal from the ECOG sensor is computed using the model of a point dipole in a homogeneous space as described by Sanz-Leon et al. (2015) (see Section 3) and the hybridLFPy (Hagen et al., 2016) software is used for computing the signal from the recording site of the implanted probes (see Section 3). The latter software uses morphologies and spatial position of neurons to generate the underlying local field potential (LFP) for given spike trains of point neurons. The morphology of neurons is taken from the presented morphology in Shuman et al. (2020). The excitatory morphology is based on the pyramidal cell morphology, and inhibitory neurons are based on the basket cell morphology (Shuman et al., 2020).

### 2.2 Output signal from the virtual experiment

**Figure 3:**
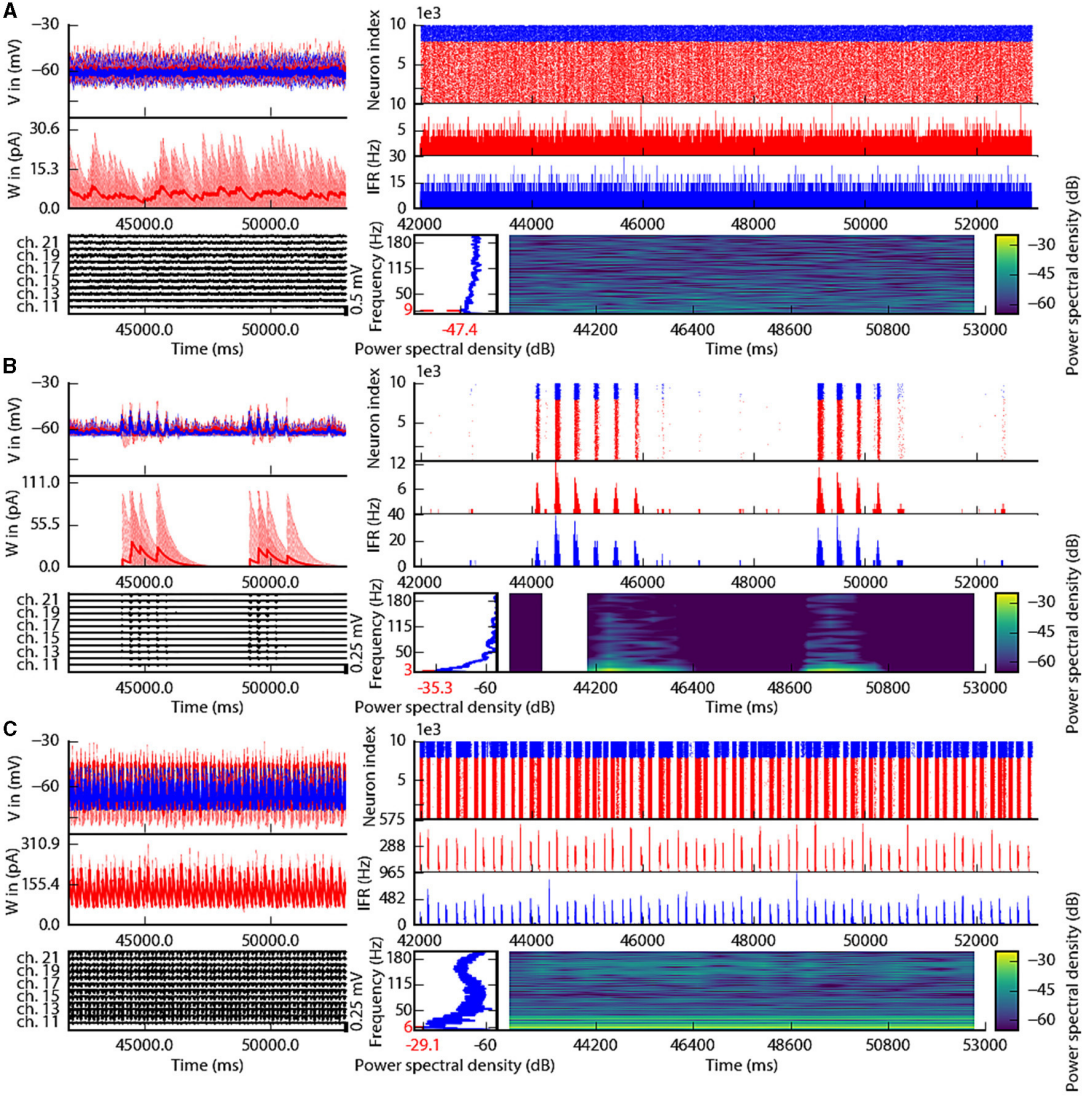
Spiking neural network in three different states of the left CA1. The parameterization of the spiking neural network of CA1 is chosen such that the dynamics are in an asynchronous state **(A)**, irregular synchronization state **(B)**, and regular bursting **(C)**. Top-left: Voltage membrane of 20 adaptive exponential leaky and integrator neurons and their mean in a thick line. The red (blue) lines are excitatory (inhibitory) neurons. Middle-left: The adaptation currents of 10 neurons and their mean in a thick line. Bottom-left: Local field potential from the 12 sites in the middle line of the left polytrode. The local field potential is computed from the spike trains of all neurons by the software HybridLFPY (Kuhn et al., 2003). Top-right: Spike trains of 10,000 neurons for 11s. Middle-right: instantaneous firing rate of the excitatory (inhibitory) population above in red (blue). Bottom-right: Spectrogram and power spectrum example of the instantaneous firing rate for 10s.

**Figure 4:**
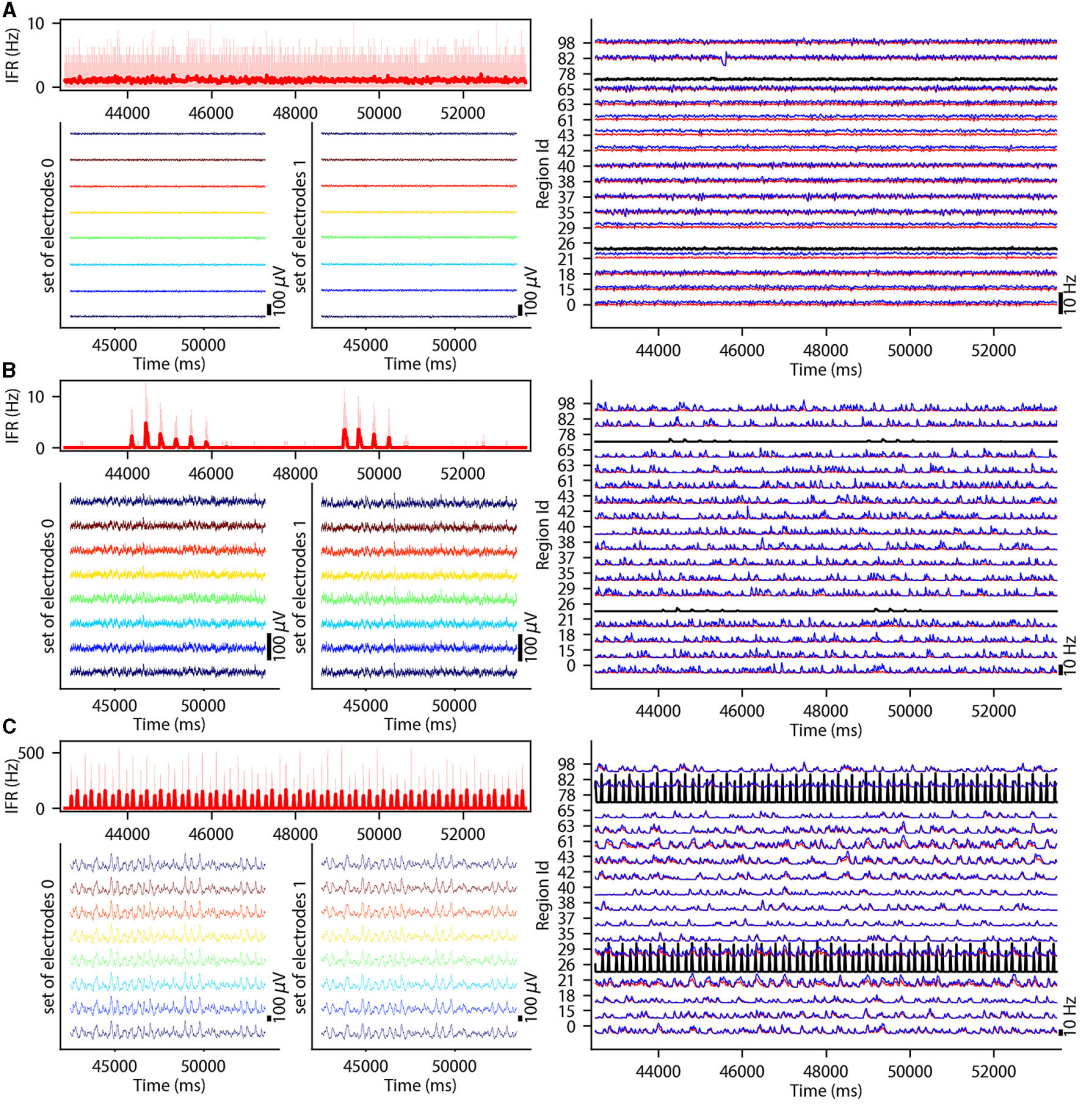
Three different states of CA1 in mouse brain. The parameterization of the CA1 spiking neural network is defined to obtain an asynchronous state **(A)**, an irregular synchronization state **(B)**, and a regular bursting **(C)**. Top-left: Instantaneous firing rate of spiking neural networks in light red for 11 s. The thick line shows the sliding window mean firing rate. Bottom-left: (bottom-right) Signal from ECOG sensors, the figure represents the recording of the 8 electrodes on the top of the left (right) hemisphere. Right part: Subset of region overview of the mean firing rates of excitatory, in red, and inhibitory, in blue, population from the model of Mean Adaptive Exponential. The two black curves are the mean firing rate of the two populations of excitatory neurons simulated with NEST (Hahne et al., 2021).

This section describes the co-simulation results at different scales by describing the possible recordings of physiological signals from the simulation of CA1 embedded in a whole mouse brain. The Discussion section will provide an interpretation of these results to describe the advantages and the limitations of the multiscale co-simulation design pattern. As described in Figure 2, the output modalities of one virtual experiment have direcly corresponding measures in the real world such as the local field potential measure at every thirty-two sites of each polytrode electrode (Figure 2J) and from the sixteen electrocorticography channels of each hemisphere (Figure 2K). Moreover, the simulation gives access directly to the voltage membranes of the CA1 neurons (Figure 2H), adaptive current of the CA1 neurons (Figure 2G), spike times (Figure 2I) and the mean firing rate of the different regions of the mouse brain (Figure 2M). To illustrate the variability of the measures and some limitations of the coupling model of different scales, we choose three sets of different parameters for CA1 and neural masses. Each set of parameters represents one of three dynamic regimes of the CA1. These results are separated between micro (Figure 3) and macro (Figure 4) scales, but they are the output of the simulation workflow between TVB and NEST. In particular, Figure 3 reports the mean voltage membrane, mean adaptive current, instantaneous firing rate and the signal of 12 central sites from the 32 electrode sites of the specific CA1 network. Figure 4 displays the results on the whole brain level: the mean firing rate of each brain region, the signal of the 16 electrocorticography channels and the mean firing rate from the spiking neural network.

To illustrate the basic dynamic features of the network, we define three operating regimes corresponding to irregular asynchronous and synchronous activity, and regular bursting. The Figures 3, 4 are separated into three different panels, which correspond to the three dynamic regimes and corresponding parameters representative of the different types of dynamics exhibited by spiking neural networks (see Section 3 for the choice of these parameters). Panel A represents an asynchronous (A) state, which is characterized by a constant (flat line) the mean firing rate (see Figure 3A top-right and Figure 4A top-left). Panel B represents an irregular synchronous (IS) state, which reflects a large irregular variation of the mean firing rate (see Figure 3B top-right and Figure 4B top-left). Panel C represents regular bursting (RB) reflecting regular oscillations (see Figure 3C top-right and Figure 4C top-left) and a second dominant high frequency (see Figure 3C bottom-right).

#### 2.2.1 Results at microscale

The top left of Figures 3A–C show the membrane voltages for ten excitatory neurons (thin red curves) and ten inhibitory neurons (thin blue curves) and mean membrane voltage of these neurons (thick curves). The middle left of Figures 3A–C represent the adaptive currents from the same ensemble of neurons (thin curves) and the mean adaptive current of these neurons (thick curves). The third biological observable from the simulation is the Local Field Potential which differs among panels (see bottom left of Figures 3A–C). The top right of Figures 3A–C display spike raster plots of the excitatory population, in red, and the inhibitory population, in blue, of the left CA1. The spiking activity is homogeneously distributed between neurons and time frames for the A state, while the other two states show co-activation of neurons with different periods. The associated instantaneous firing rate is shown in the middle right of Figures 3A–C. The spectral analysis of the instantaneous firing rate displays a peak around 3 Hz for the IS state (bottom left of Figure 3B), no peaks for the A state (bottom left of Figure 3A), and two peaks (around 6 Hz and 160 Hz) for the RB state (bottom left of Figure 3C). For the RS state, the frequency of the first peak, 6Hz, is also present in the mean of the adaptive currents, while the second peak is associated with the burst time, as shown in further detailed in Supplementary Figure 1.

#### 2.2.2 Results at macroscale

The top left of Figures 4A–C display the instantaneous firing rate (light red) of the spiking neural network with the associated transferred mean firing rate of the left region of CA1 (thick red line). The neural network's different states affect the ECOG signals, as shown in the bottom left of Figures 4A–C. The mean firing rate of excitatory (blue) and inhibitory (red) populations of each brain region are plotted in the graph on the right part of Figures 4A–C and Supplementary Figures 2–4.

### 2.3 Workflow between NEST and TVB

The previous multiscale example uses the workflow between TVB and NEST for the co-simulation. As an implementation of the design pattern, this workflow comprises five modules: two simulators (TVB and NEST), one launcher and two transfer modules. All these modules are built with the capability to be repurposed or replaced, allowing for adjustments of components of transfer modules or communication protocols (see Discussion). Two additional proofs of concept were implemented to demonstrate the possibility of the reusability of the components. The first example replaces NEST with NEURON, and the second replaces TVB with Neurolib (see Supplementary Figure 25). Moreover, without extra development, we get a proof of concept of co-simulation between NEURON and Neurolib.

The simulators perform the actual integration of the dynamics in time and require two properties to be integrated within one optimized and coherent workflow. The first property is time delay equation management, essential for reducing data transfer overhead. The second property is the presence of a high bandwidth Input/Output (I/O) interface that facilitates the efficient exchange of data and parallel execution of the simulators. Since TVB and NEST did not have generic high bandwidth I/O interfaces by default, these had to be implemented for each simulator. Details of how these I/O interfaces were created are reported in Supplementary File 1. Briefly, the NEST interface uses the device nodes with a specific back-end, while TVB uses proxy nodes which are the interface with the external software.

**Figure 5:**
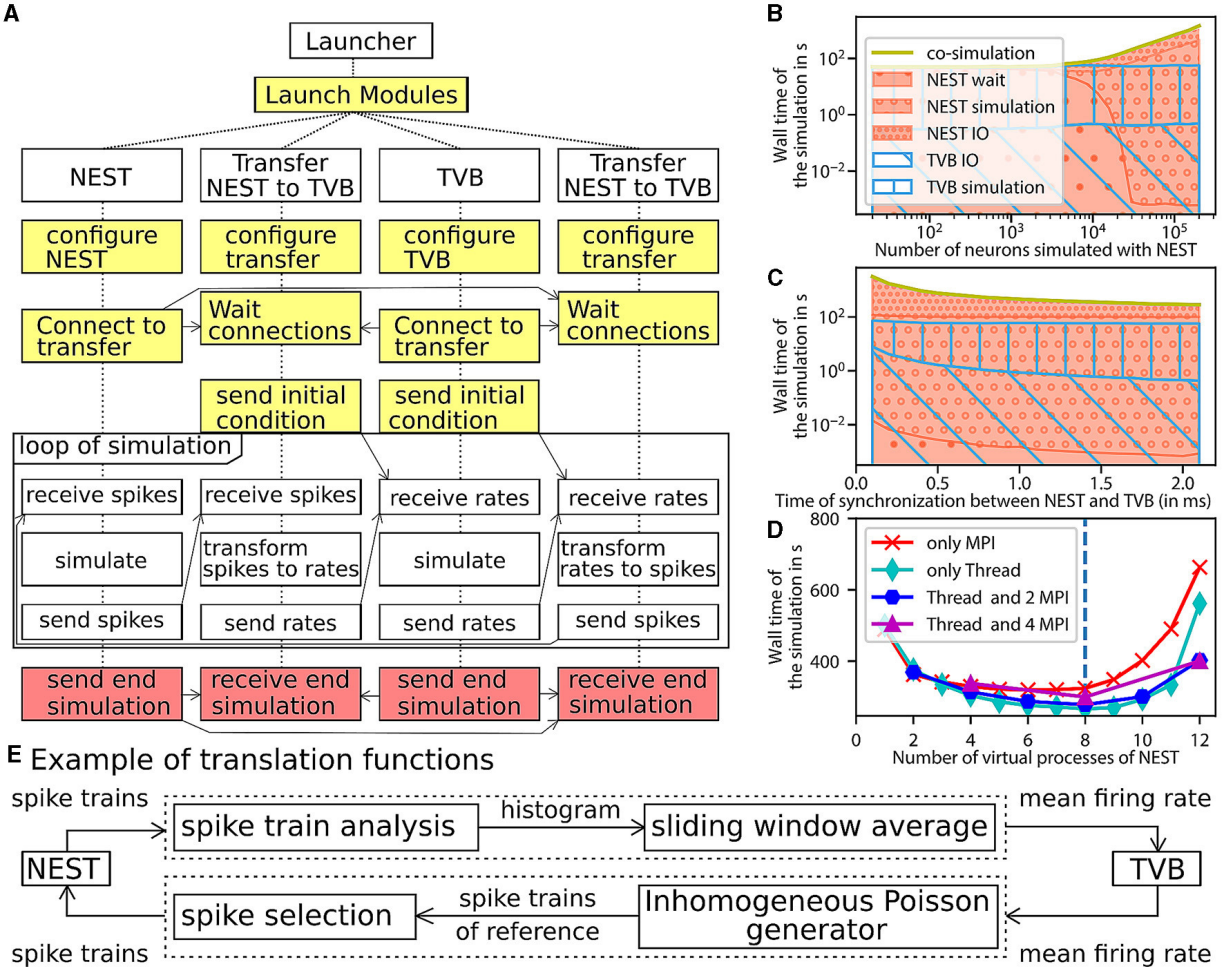
Architecture and performance of the co-simulation. **(A)** The interaction among the modules and data exchanges during co-simulation execution. The boxes in yellow mark start-up: initialization and configuration, the boxes in red for the termination of the simulation and the boxes in white for the simulation phase. **(B–D)** Performance of the workflow is obtained for 1 s of simulated time (see Section 3 for more details). The reference implementation use 1 MPI process, 6 virtual processes/threads, a synchronization time step of 2.0 ms, and simulates 20,000 neurons. **(B)** The wall clock time of the simulators as a function of the number of neurons. The total time of the co-simulation is represented in yellow. The “wait”, “simulation”, and “IO” times of NEST are represented in red surface with respectively hatches with big circles, small circles and points. The “simulation” and “IO” times of TVB (Sanz Leon et al., 2013) are represented in the blue surface with respectively hatches horizontal lines and oblique lines. **(C)** Simulation time depending on the synchronized time between simulator. The color code is the same as the **(B)**. **(D)** Wall clock time depending on the number of virtual process used by NEST (Hahne et al., 2021). The green, blue, purple, red curves are associated with different parallelization strategy of NEST, respectively, only multithreading, 2 MPI processes with threads, 4 MPI processes with thread, and only MPI processes. The vertical blue line represents the number of cores of the computer. **(E)** The “transform between spikes to rate” and “transform between rates to spikes” blocks are displayed with the different steps for transformation of data between TVB and NEST.

The launcher prepares the environment for the simulation and initiates all the other modules, as shown in Figure 5A (see details in the Supplementary Figure 5). The preparation consists of creating folders for the different modules, the logger files, and the common file with all the parameters of the co-simulation. Creating the parameters file provides the functionality to enforce consistent constraints on the parameters to be shared between the modules, such as ensuring the same integration step in both simulators, which is needed for correct synchronization between modules.

The transfer modules connect simulators by transferring data between scales and adapting the communication delay throughout the simulation. Each module is comprised of three components: two interfaces and one transformer (see Figure 1A, Supplementary Figure 14). These components are implemented in different files for reusability and modularity and are tested independently to ensure robustness (see Supplementary Figure 12). The interfaces are specific to each simulator, while the transformation can be extended, modified or reused since the transformation function is implemented as an independent process (see Supplementary File 2).

The components exchange data using a simple Application Programming Interface (API). The API is based on four functions and assumes that the connections are already established. The functions are “check if ready to get or send data”, “transfer data”, “end the transfer” and “release the connection” (see Supplementary File 2, Supplementary Figure 15). The API is implemented with two different technologies depending on the nature of the parallelisation of the components (multiprocessing or multithreading). In the case of multiprocessing, each component runs in an individual process, and a Message Passing Interface (MPI) is used to transfer data. In the case of multithreading, each component runs in an individual thread in a shared process, and the data is transferred using shared memory. Multithreading uses fewer computational resources (see Supplementary Figure 20). The transformation function provides neural mass firing rate values by using a sliding window, shown in Figure 5E. The panel also illustrates the inverse transformation from the mean firing rates to spike trains using a multiple interaction process (Kuhn et al., 2003).

The modular workflow execution is composed of three main blocks: start-up, simulation-loop and termination (see Figure 5A and details in the Supplementary Figure 5).

The start-up procedure allocates a logger for each component, facilitating debugging of the co-simulation. Subsequently, the modules and their communication channels are configured according to the parameter file. At this stage, several initialisation files are generated with simulation parameters only available after instantiation of the model (e.g. id of NEST devices and MPI port description). The details of the generation of these files are described in Section 3.2.1.

Once the simulation is launched, the simulator time clocks are synchronized using asynchronous message passing: At each multi-simulator synchronization step, the simulator receives input data via an asynchronous message in MPI, after which the next step is simulated. The transfer modules can buffer data for one synchronization step until the receiving simulator is available for receiving. Each simulator requires an initial condition (NEST: initial voltage membrane and adaptation current and TVB: state of the node during the previous seconds) and an initial message. For TVB, this initial message is sent by the transformer processes while, for NEST, it is produced by transforming the initial condition of TVB.

Ultimately, the termination occurs at the end of the simulation by the simulators themselves (see Section 3 for details).

### 2.4 Performance

The evaluation of the performance is made against a fictitious workflow with optimal performance, a co-simulation with instantaneous communications between simulators. As all the modules are designed to run in parallel, the co-simulation time for each module is identical and equal to the total running time. The focus is only on the simulator timers because the time of the transformer components is dominated by the waiting time of data (see Supplementary Figure 5). The total running time of the simulators is divided into five parts. The “initialisation” time is the time of configuring the simulators and creating connections. The “ending” time is the time of closing the connections, stopping the simulator engine and terminating processes. The “simulation” time is the total time of the internal computation of simulator engines. The “wait” time is the total duration of waiting time for access to the data to transfer by the simulator interface of the transformer module. The “IO” time is the total duration of functions for exchanging data between simulators and the transfer modules minus the “wait” time.

A perfect co-simulator has the time of the slowest simulator X; thus, “wait” and “IO” times equal zero. From Figure 5B and the Supplementary Figure 17, the actual implementation is close to ideal when the number of neurons simulated by NEST is lower than 1000. In this case, TVB is the slower simulator, and NEST spends most of the time waiting for data from TVB.

When the number of simulated neurons is between 1, 000 and 20, 000 neurons, “simulation” time of TVB is approximately the same as the sum of “simulation” time and “IO” time of NEST. In this condition, each simulator is waiting for the transformation of the data among scales.

When the number of simulated neurons is higher than 20,000, NEST is the slowest simulator. In this case, the co-simulation time is determined by the “simulation” time and the “IO” of NEST. The “wait” time is zeros, and the “IO” time is higher than the “simulation” time (see Supplementary Figures 17, 21). The two principal causes are that the communication between modules is slower than inside the modules and the increased dimensionality of the input to NEST (Weidel et al., 2016) (the increase of the number of neurons increasing the size of the neural spike data because each neuron in NEST receives an individual spike train). A closer look at the performance shows that the communication spends most of the time sending individual spike trains to NEST (see Supplementary Figure 24). However, the data size is related to the model chosen and can be reduced.

As shown by Figures 5C, D, some optimisations can be implemented to reduce the problem of overhead time of communication (Weidel et al., 2016). Figure 5C and Supplementary Figure 18 represents the time delay between brain regions when delayed data is aggregated to reduce the “IO” time and, hence, the co-simulation time. In this case, the simulators are not synchronized at each time step but at n time steps (limited by the model of connection). This aggregation can reduce co-simulation time by a factor of 6 (see Supplementary Figures 18, 22). Figure 5D and Supplementary Figure 19 represent a reduction in co-simulation time by reducing the “simulation” time of the longest simulator. The increase in NEST's resources does not modify the “IO” time until the resource is available. Since the tests are running on one computer, increasing resources for NEST increases the “simulation” time of TVB and reduces the “simulation” time of NEST. However, by deploying the workflow on high-performance computing facilities, the latter result does not replicate, and the simulation time gives similar a result with an increase in “IO” and “simulation” time because the communication between nodes is slower (see Supplementary Figure 23). The second reason for this difference is the usage of multiprocessing for the transfer modules (see Supplementary Figure 20). In your implementation in Python, the multithreading is unstable on a supercomputer due to the global interpreter lock of Python (for more details, see the Section 3.4.1). Additionally, multiprocessing compared to multithreading, has the potential to distribute the different components of the transfer modules on different nodes.

## 3 Materials and methods

The simulation details and models' parametrisation are in Supplementary Table 1. The format of this table is drawn from the proposition of Nordlie et al. (2009) for spiking neural networks. This new format includes the description of brain network modeling, the description of the coupling between scales and the description of the measurements of the simulation. This format contains more details than the proposition of Nordlie et al. because it contains all the parameters for the co-simulations.

The following text provides an overview of the models, communication between modules, details of the performance tests and implementation details.

### 3.1 Models

#### 3.1.1 CA1 model

The spiking neural network of CA1 comprises two regions (left and right), which contains two populations, 8,000 excitatory neurons and 2000 inhibitory neurons. This network is simulated by NEST (Hahne et al., 2021), a neuro-informatics platform for spiking neural networks. The adaptive exponential integrate and fire neurons (Brette and Gerstner, 2005) are connected by exponential conductance-based synapses with a connection probability of 5% inside the region. The excitatory population establishes normalized weighted connections with the other regions defined by the mouse connectivity atlas. Additionally, we assume that each neuron has the same unique number of synaptic connections from other brain regions; the mouse connectome defines the repartition of these synapses. Transmission delay between regions is defined as the ratio of the distance between the regions and the transmission speed. Calculating these ratios is part of the configuration of The Virtual Brain (TVB) (Sanz Leon et al., 2013) because the data required by TVB is the track lengths between regions and the speed of the transmission. Within a region, the synaptic transmission delay is instantaneous, which is implemented in NEST by setting the delay to the smallest transmission delay supported. In addition, the neurons can receive external noise input modeled as an independent Poisson process in addition to the external stimuli received from other regions through the transfer of mean firing rates as transformed spike trains.

#### 3.1.2 Mouse brain model

The mouse brain model is simulated using The Virtual Brain (Sanz Leon et al., 2013; Melozzi et al., 2017), a neuro-informatics platform for connectome-based whole-brain network modeling. The “connectome” used here is extracted from Allen Mouse Brain Connectivity Atlas (Oh et al., 2014) in 2017. The large-scale brain network is comprised of linearly coupled neural mass models. Specifically, the model representing each region is a second-order Mean Ad Ex model (di Volo et al., 2019) with adaptation, representing the mean firing rate for an ensemble of one excitatory and one inhibitory neuronal population.

#### 3.1.3 Electrophysiological monitoring model

The electrophysiological monitoring variables are computed using two models representing the cortical and implanted sensors. The electrocorticography model is a simple forward solution of a dipole at the region level. The electric field recorded by the virtual sensors at the brain level is based on two assumptions: considering the brain as a homogeneous space, and the field is generated only from excitatory populations. With these assumptions, the recorded field is the sum of excitatory population activities, i.e. the mean excitatory firing rate weighted by the distance between the sensors and the region's center (Sanz-Leon et al., 2015). The implanted sensors' signals are computed from point-neuron activities using a hybrid scheme for modeling local field potentials (LFP). Specifically, each potential is simulated using hybridLFPy (Hagen et al., 2016), which incorporates the recorded spike from the network and the morphology of the pyramidal and basket cells.

#### 3.1.4 Choice of three sets of parameters

Three parameter regimes were implemented to simulate well-known characteristic neural network dynamics: irregular synchronous, irregular asynchronous, and regular bursting.

The parameters for Irregular Synchronous state follow the work of di Volo et al. (2019). The coupling between regions and the noise is tuned manually to the regime of fluctuations of the firing rate in each region. The Asynchronous state was realized by reducing the degree of fluctuations. The result is a reduction of the spike-triggered adaptation of the excitatory neurons, a reduction of the number of connections between the regions, an augmentation of the inhibitory synaptic weights, a reduction of the variance of the noise and the addition of a Poisson generator for the spiking neural network. The Regular Bursting state is obtained when changing the voltage reset of the membrane and the leak of the reversal potential of the excitatory and inhibitory neurons, the spike-triggered adaptation and the time constant of the adaptation current of excitatory neurons. An empirical exploration of the models is done to get a balanced spiking neural network and the desired brain dynamic. The result of this exploration is a reduction of the connection between regions and a reduction of the connection between excitatory and inhibitory neurons, a reduction of the number of connections between brain regions and a reduction of the noise variance.

All the numerical values of the parameters are in Supplementary Table 1.

### 3.2 Communication between modules

#### 3.2.1 Initialization of communication

During the initialization of the simulation, the launcher creates a specific folder for each module and an extra folder for the logger file of all components. Subsequently, the launcher creates user-defined relationships between parameters, such as copying one parameter into another to have the same values or the result of linear functions of parameters. All these parameters are saved in a file and organized in sections dedicated to a module or part of the co-simulation. The launcher also saves the initial message sent to TVB.

Once each module is launched, they will create some files in the folders generated by the launcher to initialize the communication. NEST will create two files with the ids of devices for recording and generating spikes, which are used by the transformer modules for sending and receiving spikes to the right devices. Transformer modules will create files containing the MPI port description which are used by NEST and the wrapper of TVB for connecting to them. TVB saves its initial conditions to allow possible reproducibility of the co-simulation.

#### 3.2.2 Synchronization between modules

The transfer modules synchronize the simulation by managing the access to its internal buffer and receiving status messages from the simulators. The receiver process receives the data and aggregates them in a buffer. Rate data do not need to be buffered when using MPI communication, they are sent or received directly to the transformer process. The data is transferred to the transformation function when the data of the preceding step are transformed and transferred to the sender process. The sender process gets the data after sending the data of the preceding step to the simulator. It can only send the data to the simulator when it is ready. In addition, the simulator needs to await data for the next step of the simulation. Given all these constraints, the transfer module assures correct transport and keeps the components synchronized. If needed the transfer module buffers data for a simulation step. The transfer module can receive and send data concurrently and translation can be performed while waiting for the slowest simulator.

### 3.3 Performance tests

The performance tests are realized with time recorders integrated at specific places in the code. These times are aggregated durations to evaluate the running time of the co-simulation in each section. This allows evaluating the time of “simulation”, “IO” and “wait” time. Each test is done for 10 trials of 1 second of simulated time for asynchronous configurations with one or two parameters (number of neurons, synchronization step, number of virtual processes of NEST, number of processes dedicated to NEST and number of nodes used by NEST) which vary per test. The results of the trials are averaged to reduce the variability of the measurements. The varied parameters of the tests are the number of spiking neurons, synchronized time between simulators and the configuration of MPI and thread of NEST. Figure 5 and Supplementary Figures 17–19 show the result of the performance test done on DELL Precision-7540 [Intel Xeon(R) E-2286M CPU 2.40 GHz * 8 cores * 2 threads, 64 GB of Ram with Ubuntu 18.04.5]. The communication between components in the transfer module was performed with the multithreading approach. Supplementary Figures 21–23 are generated using the Jusuf system (https://apps.fz-juelich.de/jsc/hps/jusuf/cluster/configuration.html) which is composed of nodes with 2 AMD EPYC 7742 2.25 GHz * 64 cores * 2 threads, 256 (16x16) GB DDR4 with 3,200 MHz, connected by InfiniBand HDR100 (Connect-X6). In this second case, the transfer module uses MPI protocol to communicate between components.

### 3.4 Implementation details

The source code of the co-simulation is open-source and contains Python script and C++ files. A singularity and a docker image are also available on singularity-hubs to replicate the figures as in the performance test. The activity diagram (see Supplementary Figure 5) describes in detail the interaction between each module and components for this specific virtual experimentation.

The implementation of Input and Output for NEST used the existing simulator's architecture and parallelization strategy. NEST has different back-ends for the input and output data, the creation of a new back-end for the communication of the data was enough for integration in the co-simulation design pattern (for more details see Supplementary File 1). For more technical details about the communication with NEST, an activity diagram (see Supplementary Figure 6) describes the communication protocol with NEST back-end. For this specific example, the states of the wrapper of NEST and the states of transfer components which communicate with NEST are described respectively by the Supplementary Figures 7, 8.

The implementation of Input and Output for TVB is different because TVB doesn't use MPI for its parallelization and it doesn't have an interface for exchanging data outside of the simulator. The creation of the interface required a modification of the simulator engine during its configuration for integrating the functions to exchange data with the transformer and a wrapper for communication with the transformer modules (for more details see Supplementary File 1). For more technical details about the communication with TVB, an activity diagram (see Supplementary Figure 9) describes the communication protocol with the TVB wrapper. For this specific example, the states of the wrapper of TVB and the states of transfer components which communicate with the wrapper of TVB are described respectively by the Supplementary Figures 10, 11.

The description of the transfer modules is partially described in Supplementary File 2 which focuses only on the interface with simulators. In addition to this note, the state of the different components are described in the Supplementary Figures 8, 11, 13. To better understand different instances and classes in this module, Supplementary Figure 14 describes all the instances and their role and Supplementary Figure 15 describes the composition of the abstract class and the simple API for communication. The communication protocol for data exchange between transfer module components differs depending on whether the parallelization strategy is multithreading or multiprocessing. In the case of multiprocessing, MPI protocol is used for data exchange. The communication protocol differs depending on the data type, as shown by Supplementary Figure 16. The spike trains are variable and large data (they can go from a few Kilobytes to more than one Megabyte depending on the firing rates). According to it, we choose to use shared memory for transferring data. For the mean rate data, the data size is constant and small (a few Kilobytes depending on the number of regions). According to it, we choose to use the functions Send and Receive of MPI protocol for transferring the data. In the case of multithreading, only a shared buffer is used between threads.

#### 3.4.1 Deadlock due to global interpreter of python

In the case of multithreading for internal communication in the transfer modules, the program may be in a deadlock because the interface with a simulator does not receive the information of receiving data. As it is explained in the global interpreter lock documentation, “The GIL (global interpreter lock) can cause I/O-bound threads to be scheduled ahead of CPU-bound threads, and it prevents signals from being delivered” (https://wiki.python.org/moin/GlobalInterpreterLock). The consequence is that some signals used by MPI are not delivered, which creates a situation where a simulator and a transformer are waiting for an MPI message from the other one, but these messages will never arrive.

## 4 Discussion

The Parallel Co-simulation toolbox presented here provides co-simulation technology linking two simulators operating at two different scales with the only two requirements that the simulator simulates time delay equations and has an interface for sending and receiving data from outside of itself. In our application, the simulator needs to use MPI to send and receive data. This workflow is based on the cyclic coupling topology of modules (Chopard et al., 2014), i.e each module regularly receives new inputs during the simulation. The two scale-specific simulators are interchangeable due to the genericity of the transfer function, as well as the modularity and design of the transfer module (for more characterization of the workflow, see Supplementary File 3). The interfaces of the simulators and other modules serve as a software science co-design pattern and can be reused in other studies involving co-simulations.

Our approach separates the theoretical challenge of coupling models at different scales from the technical challenge of coupling the corresponding simulators. The simplicity of the design pattern allows the scientific community to advance their research project without being hindered by technical details. Best practices are advised on carrying out a task or implementing the design pattern. These challenges are not unique to using the Parellel Co-simulation toolbox, but apply to most technical implementations of multiscale modeling software. On the technical side, the design pattern does not provide guidelines for the co-simulation's robustness, management and maintenance, similar to the closely related staged deployment and support software for multiscale simulations developed in EBRAINS (https://juser.fz-juelich.de/record/850819). On the conceptual side, for proper use of co-simulation technology, a profound understanding of the involved models is necessary to avoid operating the models outside of their valid parameter ranges. For instance, the neural mass model used in this paper cannot capture the fast scale dynamics, especially the fast regimes of regular burst state (RS) (Boustani and Destexhe, 2009). In the neural mass model's derivation, the input firing rate of the neurons is assumed to be an adiabatic process, which is valid in some parameter regimes, but violated for the irregular synchronous state (IS), in which rapid transitions between low and high firing rates occur. As co-simulation requires an understanding of models typically used in at least two different and non-overlapping fields, particular attention should be paid to the responsible use of multiscale models. Such caution should also be applied here when interpreting the results of the CA1 model and the full brain network model used in this paper. Numerical errors constitute another issue. As these errors cannot be estimated analytically, the alternative solution is to perform a sensitivity analysis or uncertainty quantification to determine whether or not the simulation result is reliable (Coveney and Highfield, 2021; Coveney et al., 2021).

For the validation of the co-simulation, it is essential to generate data that can be related to real-world observations, as is the case here with the model of the two types of electrodes. A critical issue is the repeatability and reproducibility of the simulations. Repeatability is ensured by managing all the random generators in each simulator and using a single parameter file for the co-simulation setup. For reproducibility, due to the complexity of the network, a table is proposed where the configuration of each simulator is reported with their version and also the description of the transformation modules (see Supplementary Table 1). A notable property of this design pattern is the independence of its modules and components. This independence allows unit testing for each of them. Our design pattern also requires the implementation of a minimal reusable simulator interface for interaction between simulators. In a possible second stage, this interface can be adapted to a standard to increase the possibility of interaction with other simulators.

EBRAINS provides two solutions for co-simulation (Schirner et al., 2022) using The Virtual Brain, that is TVB-Multiscale tool and the here described Parallel Co-simulation toolbox. The two tools implement conceptually and technically two different solutions. The TVB Multiscale tool focuses on user convenience, allowing for rapidly prototyping scientific use cases using a single interface to configure all modules in the co-simulation. It is based on serial approaches for the co-simulation, i.e., each module is run one after the other. The Parallel Co-simulation tool, on the other hand, focuses on optimizing performance. The detailed description, benchmarking and validation of the Parallel Co-simulation toolbox is the topic of the current manuscript. Consequently, the TVB-Multiscale tool is slower. Performance tests show that the various modules run in parallel and adapt to the slowest module (see Section 2.4). The waiting time of the slowest modules is quasi-null, which means there is no loss of time in the synchronization of modules. Performance in co-simulation is an important criterion, as the microscopic simulators are typically very high-dimensional and hence computationally costly. The serial approaches can be interesting when a computer does not have at least one CPU core per module because, under this condition, modules need to share resources which can slow the co-simulation. This was demonstrated by the large increase of simulation time when the number of virtual processes for NEST is higher than the number of physical CPU cores. The other important distinguishing feature of the two co-simulation toolboxes is the unique interface for multiscale simulations. Similarly to TVB-Multiscale, multiscale simulators have the advantage of having a unique interface for multiscale simulations. This unique interface simplifies the simulation configuration but also reduces the specificity of functionality for each scale, which may be disadvantageous for some situations, such as optimization. For example, in our application, spiking neuron and brain region models require different integrators to avoid numerical errors and enhance efficiency. The CA1 model is a sparsely connected network of thousands of neurons using event communications. The mouse brain is a fully connected network of hundreds of regions based on continuous communications. Consequently, the optimization strategy is different and requires specificity.

Other existing frameworks to deploy and communicate runtime data between simulators comparable to Parallel Co-simulation include the Multi-Simulation Coordinator (MUSIC) (Djurfeldt et al., 2010). By default, MUSIC does not include modules that facilitate translation between scales, which is needed when coupling simulators on different scales of abstraction. An extension of MUSIC has been proposed in Jordan et al. (2019) which proposes encoders and a decoder for transforming data and adapters for connecting to other systems of communication, such as ZeroMQ (Hintjens, 2013) and ROS (Quigley et al., 2009). The design of this extension has some similarities to our design pattern (Weidel et al., 2016) and allows easy extension to include new methods in the future. However, the main difference with this extension is the parallelization of modules. On a more technical level, a second difference is how MUSIC uses the HPC transport protocol Message Passing Interface (MPI) (Message Passing Interface Forum, 2015). MUSIC takes ownership of the highest level MPI environment (MPI_COMM_WORLD); this can cause challenges when integrating MUSIC with simulators that expect exclusive ownership of this highest level. Our implementation does not touch this highest-level ownership. We use MPIs client-server functionality to connect between simulators, completely evading this challenge. This difference in MPI usage also allows better use of the HPC scheduling mechanisms as each simulator is deployed in isolation, facilitating optimal workload placement on the hardware available. MUSIC does support several features currently not implemented in our implementation of the design pattern: multi-rate integration, i.e., different frequencies of sending and receiving data from simulators, generic configuration file and it prevents some simulation errors by using the MPI error system. On the other hand, our implementation of the design pattern allows for easy extension with new simulators and better distribution on HPC systems.

Outside of neuroscience, standards exist for co-simulation, such as High Level Architecture (HLA) (Saunders, 2010) and Functional Mock-up Interface (FMI) (Andreas Junghanns et al., 2021). These standards include error management, multi-rate integration, and data management (Saunders, 2010; Blockwitz et al., 2012). The main difference between MUSIC and our design pattern with these two standards is the communication strategy between simulators. FMI provides a standard for exchanging models and for scheduled execution (Andreas Junghanns et al., 2021). Features of FMI and MUSIC currently not implemented in our design pattern are: Real-time hardware interactions (Moren et al., 2015; Andreas Junghanns et al., 2021). Additionally, FMI supports signal extrapolation for error reduction (Blockwitz et al., 2012), although this could be added in the translation modules central in our design pattern. FMI does not support concurrent execution of the different simulators, although internally, the simulations can be parallelized (Andreas Junghanns et al., 2021) and FMIGo proposes a parallelization implementation of FMI (Lacoursire and Hrdin, 2017). HLA is designed for distributed systems and provides a standard for data exchange and time management of simulators (Neema et al., 2014; Gutlein et al., 2020). HLA facilitates the re-usability and interoperability of simulators and models by describing each component's roles and interactions. It further formalizes the data exchange and coordination between simulators and follows the publish/subscribe pattern. This standard specifies the definition of information produced or required by simulators. It provides a common data model for the reconciliation of model definitions and interoperability of simulators including during distributed runtime execution. Typically these services are implemented with a centralized communication architecture (Gutlein et al., 2020), sometimes described as a hub and spoke model. Both MUSIC and our design pattern use direct, peer-to-peer communication.

In addition to the simulator interfacing standard, there are standards more focused on coupling models at different scales, such as MUSCLE2 (Borgdorff et al., 2014), Yggdrasil (Lang, 2019) and Vivarium (Agmon et al., 2022). This different standard provides solutions to the semantic and syntactic problems induced by multi-scale issues that are not addressed by our design pattern. Our design scheme is model-independent and delegates responsibility for the consistency of multi-scale modeling to users. However, one of these standards can be merged to provide a complete digital platform for multi-scale co-simulation.

In summary, we have presented a new software science co-design pattern of the Parallel Co-simulation tool for coupling simulators with a transformation module. This design pattern provides the first step for developing platforms using transitional scaling models and structuring the future syntactic, semantic and conceptual issues induced by multiscale problems. The optimization for this workflow is based on the communication delay between scales. It is not generalized for all cases but recommended for models with transmission line element method (Braun and Krus, 2016) or waveform relaxation method (Nguyen et al., 2007).

## Data availability statement

The datasets presented in this study can be found in online repositories. The names of the repository/repositories and accession number(s) can be found below: http://dx.doi.org/10.5281/zenodo.7259022. The co-simulation between TVB and NEST is freely available under v2 Apache license at https://github.com/multiscale-cosim/TVB-NEST on the branch Paper TVB-NEST and Paper TVB-NEST with timer. A docker container containing the project is freely downloaded on Ebrains (https://docker-registry.ebrains.eu/harbor/projects/53/repositories).

## Author contributions

Conceptualization: LK, SD-P, and WK. Methodology and investigation: LK, SD-P, WK, and KS. Visualization and writing—original draft: LK. Supervision: AM and VJ. Writing—review and editing: LK, SD-P, WK, KS, CB, and VJ. All authors contributed to the article and approved the submitted version.
