# Supplementary material for: Multiscale co-simulation design pattern for neuroscience applications
Source: Front Neuroinform. 2024 Feb 12;18:1156683. doi: 10.3389/fninf.2024.1156683 (PMC10895016; doi:10.3389/fninf.2024.1156683)
Supplement: Supplementary file 1 [file Data_Sheet_1.pdf]

Supplementary Material

Supplementary Material	Title
Supplementary Note 1	Guidelines Input/Output (I/O) interface
Supplementary Note 2	Guideline for implementation of transfer module
Supplementary Note 3	Detail characterization of the workflow TVB-NEST
Supplementary Tabular 1	Tabular describing the co-simulation
Supplementary Figure 1	Zoom on 1s for the spiking neural network with regular bursting state
Supplementary Figure 2	Mouse Brain activity for Asynchronous Irregular
Supplementary Figure 3	Mouse Brain activity for Synchronous Irregular
Supplementary Figure 4	Mouse Brain activity for Regular Bursting
Supplementary Figure 5	Detail sequence diagram of the co-simulation
Supplementary Figure 6	Sequence diagram of the communication protocol with NEST
Supplementary Figure 7	State diagram of NEST wrapper
Supplementary Figure 8	State diagram of transfer components for interaction with NEST
Supplementary Figure 9	Sequence diagram of the communication protocol with the wrapper of TVB
Supplementary Figure 10	State diagram of wrapper of TVB modules
Supplementary Figure 11	State diagram of transfer components for interaction with the wrapper of TVB
Supplementary Figure 12	File organisation of the transformer module
Supplementary Figure 13	State diagram of transfer components which transform data from one scale to another
Supplementary Figure 14	Structure diagram of the two transfer modules with the description of each component
Supplementary Figure 15	Class diagram of the transfer modules
Supplementary Figure 16	Communication between components in the transfer module
Supplementary Figure 17	Details of the performance with the increase of neurons
Supplementary Figure 18	Details of the performance with the increase of synchronize time
Supplementary Figure 19	Details of the performance depending on the number of processes and threads for NEST

Supplementary Material	Title
Supplementary Figure 20	Compare the performance depending on parallelization strategies of the transformer modules
Supplementary Figure 21	Performance of the co-simulation on one supercomputer for different number of neurons
Supplementary Figure 22	Performance of the co-simulation on one supercomputer for different time of synchronization between simulator
Supplementary Figure 23	Performance of the co-simulation on one supercomputer for different number of node for NEST
Supplementary Figure 24	Details of the timer of one run for the reference configuration
Supplementary Figure 25	Proof of concept of replacing NEST and TVB by other simulators

1 SUPPLEMENTARY NOTES

SUPPLEMENTARY NOTE 1| GUIDELINES INPUT/OUTPUT (I/O) INTERFACE

Before creating the I/O interface, the simulator must be analysed. The objective is to verify the existence of output and input devices, the paradigm of parallelisation, the tools for the parallelisation and the properties to keep. This analysis will help to modify the architecture of the simulator. The modification needs to follow the simulator development and its maintainability. The last part is the creation of a wrapper to communicate with the transfer module if required. Two paragraphs will provide further details about how to transfer data from NEST [1] to TVB [2] and vice versa.

NEST I/O interface

NEST has two types of devices: stimulating and recording devices. These devices receive or send messages mainly based on spikes times. The parallelisation uses MPI and/or threading depending on its parametrisation, and it is based principally on event transfer (spikes between neurons). The critical property to conserve is its scalability. From this statement, a new interface was implemented into version 3 of NEST and uses MPI communication. The modification architecture of NEST is creating a specific back end of the recording and stimulating devices and reformatting input devices to include the usage of a specific back end. Each back-end uses a particular communication protocol (see supplementary figures 6), which includes transmitting the NEST state using tags and transferring data. The transfer module directly uses this interface (see supplementary figures 13).

TVB I/O interface

The simulator engine is a class composed of different classes for the simulation of Brain network modelling. The I/O interface present in TVB is the ”monitor” classes for recording and the ”stimulus” classes stimulating. The ”monitor” classes record only the data in memory and are limited to some recording values. The ”stimulus” classes have the particularity that the stimulus requires to be defined by an equation at the beginning of the simulation. For the parallelization paradigm, TVB does not have a strategy of parallelization. We do not identify specific properties to keep for the optimisation co-simulation.
From these statements, the prototype uses a new monitor that dynamically modifies the simulator. That means that the new monitor, during its instanciation, modifies the instance of the simulator to include new functions and parameters used for the co-simulation. The

new parameter added by this new monitor is an extra buffer to delay the simulated data. The new functions are the I/O interface. The output recording of the stimulation of the nodes due a network connections and the input is the state of some nodes. The recording and the integration of the data from the new I/O interface are based on the usage of some nodes in the network as proxies. This means that the state of these proxy nodes is defined by external data and not a mean-field model. The receiving stimulation of these proxy nodes by the network is recorded and transferred by the new I/O interface. Following the simulator development and its maintainability, this new monitor is not included in the code of TVB. The main reason is that it is difficult to maintain and debug dynamic modifications (it requires instantiation of the object for debugging and the code of the object is in two files). The current official release of TVB includes a new class of 'co-simulator', a sub-class of simulator engine, that implemented the I/O interface.
However, this interface is not enough for communication with the transfer module in our application because there is a need to communicate data with MPI communication. A wrapper around this I/O interface is implemented to overcome this requirement(see supplementary figure 10 for details). A bug present in the implementation of this new monitor does not take into account the time of synchronisation between the simulator, but it does not have an impact on the co-simulation dynamic.

SUPPLEMENTARY NOTE 2| GUIDELINE FOR IMPLEMENTATION OF TRANSFER MODULE

This section focuses on the intention behind the implementation of the transfer modules. In the future, two types of scientists will improve and use transfer modules. The neuroscientist or physician will modify it to create new models and adapt them to their scientific questions. In parallel, computational scientists will work to improve communication speed between all modules and components. Furthermore, in the future, there will be a need to add other simulators such as Neuron[19], Arbor[20], Neurolib[26]. The architecture design needs to simplify the addition of other simulators and other types of data (membrane voltage, current, ...).
The separation of the neuroscience research and computer science research is done by the separation of the functions of the transfer module in three components/objects/processes: two for the I/O interface with simulators and one for transformation functions (see Supplementary Figure 14). A neuroscientist will principally modify the transformation components where the meaning of the data transformation is required and important for his work. A computational scientist will focus on optimising the communication with the

interface with a simulator, the internal communication and the management of the data flux. To avoid conflict between this type of research, there is a simple API for receiving and sending data in each component (see examples of activity diagram of the transfer components in the Supplementary Figure 16). The only constraint to the neuroscientist is to respect the buffering of data in the transformation function by releasing the input connection before accessing the output connection. Moreover, this simple API is implemented following the abstract factory pattern. This design pattern is chosen to help the comparison of different implementations of communication and the integration of new simulators.
The API address partially the constraint of the simplification for adding a new simulator because only one missing part is a component for the interface with the simulator; the rest can be reused. The other architecture element for this constraint is separating files for each simulator and encapsulating the interface in an abstract class following a composite pattern. The second constraint is the simplification of adding a new type of data respected by the imposition of a convention for data management. This convention comprises four functions and one Boolean for sending and receiving data. The functions are ”ready for transfer data?”, ”transfer the data”, ”end of transfer data” and ”release the connection”. The Boolean contains information about the connection statement from the other side (0:open or 1:closed).

SUPPLEMENTARY NOTE 3| DETAIL CHARACTERIZATION OF THE WORKFLOW TVB-NEST

This characterisation is based on the taxonomies proposed in Gomes et al. 2018 [3]. However, this taxonomy is not the best for this workflow because the transformation modules are not considered. Additionally, one of the hypotheses of this taxonomy is the presence of an orchestrator, which is not the case for the workflow.

Non-Functional Requirements

- Fault tolerance: No (NEST does not store the previous state and the communication spike, which creates the impossibility of coming back in the future)
- Configuration reused: Yes (the configuration of each simulator is independent and defined during the initialisation)
- Performance: Yes and No (the simulator’s scalability and parallelisation are kept, but there is no modulation of the integration step or signal extrapolation).
- IP Protection: No protection (NEST and TVB do not use protected models.)

- Parallelism: Yes (the communication use MPI and each simulator is run in individual processes)
- Distributed: Yes (the workflow keeps the properties of NEST to be simulated in a distributed way)
- Hierarchy: Yes (the workflow is independent of the model for each simulator and the transformation function. There is some requirement for the connection between modules which creates the dependencies.)
- Scalability: No (it is dependent on the simulators)
- Platform independent: Yes and No (it requires some dependence on the platform, but the usage of docker or singularity can pass it)
- Extensibility: Yes and No (some extra modules such as NESTML or TVB can create models for each simulator but not a specific extension for the transformation and all the simulations.)
- Accuracy: No (there are any simulators which provide the errors or the convergence of the simulations.)
- Open Source: Yes (each simulator is open source, and the workflow is also open source)

Simulator Requirements

Information Exposed

- Frequency of State: No (the frequency of the state for the simulator and the co-simulation is fixed during the initialisation)
- Frequency of Outputs: No (same as before. Moreover, TVB can have an output frequency lower than this internal integration frequency)
- Detailed Model: Yes (the code for all the models is available)
- Nominal Values of Outputs: dependent on the output and the models used
- Nominal Values of State: dependent on the model
- I/O Signal Kind: No (there is not a master algorithm but NEST has some internal statement about the signal communication between devices and nodes.)
- Time Derivative: Output only
- Jacobian: No
- Discontinuity Indicator: No (the transformation modules handles this part)
- Deadreckoning model: No
- Preferred Step Size: No (the step size is fixed at the beginning)

- Next Step Size: No (there is not an orchestrator for managing the step size and the step size are fixed)
- Order of Accuracy: No (there is no extrapolation function)
- I/O Causality: Propagation Delay (the delay is used for the parallelization. However this delay is fixed during the simulation)
- Input Extrapolation: No (there is no extrapolation function)
- State Variables: Values
- Micro-Step Outputs: Yes (TVB and NEST give the output of each micro-step but it can be modulated)
- Worst Case Execution Time: Yes (the worst case is when the minimum delay is equal to the micro-time step (see Performance section))

Causality

Causal

Time Constraints

- Analytic Simulation: False (there does no analytic solution to this co-simulation)
- Scaled Real Time Simulation: Fixed for TVB and NEST
- Rollback Support: No (there is no rollback support for NEST and TVB)

Availability

local

Framework Requirements

- Standard: No standard (ad-hock solution)
- Coupling: Input/Output Assignments (Transformation modules between the two simulators take the role to synchronize the I/O of the simulators)
- Number of Simulation Units: Two simulators
- Domain: Hybrid
- Dynamic structure: No (all the dependency is defined at the beginning)
- Co-simulation Rate: Single (unique size of the synchronization step between simulators and micro-step is fixed during the simulation)
- Communication Step Size: Fixed

- Strong Coupling Support: None – Explicit Method (the transformation module contains the information on the coupling of the simulators)
- Results Visualization: It can be in live or postmortem
- Communication Approach: Jacobi (however, the delays allow the separation of micro-steps without creating errors)

Additional : characterization of the coupling[4][5]

The previous characterization is focusing more on the technical details but it is missing the characterization of the transformation modules. For the workflow of TVB-NEST, the scales are separate in space (micro- and macro-scale). The coupling between the simulators is a tightly coupled or cyclic coupling using a fixed number of simulators instance. The workflows allow sequential or parallel execution depending on the number of initial conditions.

2 SUPPLEMENTARY TABLES AND FIGURES

2.1 Table

A1	Co-simulator environment	
Simulator	NEST[1]	TVB[6]
Version	3.0	2.0
Integrator method	4th order Runge-Kutta-Fehlberg method	Heun method
Integration step size	0.1 ms	0.1 ms
Synchronization time step	2.0 ms	
Simulated time	60.0 s	
Analyzed time	between 42.5 s and 53.5	
Type of I/O interface	proxy input	proxy region

A2	Co-simulator architecture	
Reference model	The mouse brain with 104 regions	
Simulator	NEST	TVB
Number of simulated region	2	102
Number of MPI processes	3	1
Number of thread per process	6	1
Number of random seeds	1	
Transfer module	NEST to TVB	TVB to NEST
number of transfer module	2	2
Number of MPI processes	2	2
Number of threads or processes	6	6
Number of random seeds	1	1

B1	NEST : Model Summary
Topology	left and right CA1 connected to TVB
Population	2 by regions : excitatory and inhibitory
Connectivity	random convergent connection
Neuron Model	adaptive exponential leaky integrate and fire neurons[7], fixed threshold and fixed absolute refractory time
Synapse Model	conductance-based exponential shape
Plasticity	--
Input	Independent fixed rate Poisson generator spike trains to all neurons and spike trains from TVB
Measurement	Voltage, Adaptation Current, Spike Activity and Model of Local Field Potential signal

B2	NEST : Topology
regions	2 regions (left CA1 and right CA1)
number of neurons by regions	N=10000
percentage of inhibitory neurons	g_{inh}=20%

B3	NEST : Population by regions	
**Name**	**Elements**	**Size**
E	aeif_cond_exp	N_e = (1-g_{inh})N = 8000
I	aeif_cond_exp	N_i = g_{inh}N = 2000
P_{ext}	Poisson generator	1
I_{ext}	spike generator (input from TVB)	N

B4	NEST : Neuron Model
Name	aeif
Type	adaptive exponential leaky integrator[7] and fire with conductance synapse
subtreshold dynamics	$$C_m \frac{dV_m}{dt} = - g_L(V_m - E_L) + g_L \Delta_T e^{\frac{V_m - V_{th}}{\Delta_T}} - g_e(t)(V_m - E_{ex}) - g_i(t)(V_m - E_{in}) - W + I_e$$ $$\tau_w \frac{dW}{dt} = a(V_m - E_L) - W$$
reset condition	For $t^{(f)} = \{t \mid V_m(t) >= V_{peak}\}$ • $V_m([t^{(f)}; t^{(f)} + t_{ref}]) = V_{reset}$ • $W([t^{(f)}]) = W([t^{(f)}]) + b$

B5	NEST : Synapse Model
Name	cond_exp
Type	post-synaptic conductance in the form of truncated exponentials
Coupling equation	$g_e(t) = \sum_{t_j^{(f)}} w_j exp\_trunc(t - t_j, \tau_{ex})$ with $w_j > 0.0$ $g_i(t) = \sum_{t_j^{(f)}} w_j exp\_trunc(t - t_j, \tau_{in})$ with $w_j < 0.0$ $exp\_trunc(t, \tau) = e^{1 - \frac{t}{\tau}} Heaviside(t)$

B6	NEST : Excitatory Neuron Model Parameters			
	case	Asynchronous	Irregular Synchronous	Regular bursting
C_m	Capacity of the membrane	200.0 pF		
t_{ref}	Duration of refractory period	5.0 ms		
V_{reset}	Reset value for V_m after a spike	-64.5 mV	-64.5 mV	-47.5 mV
E_L	Leak reversal potential	-64.5 mV	-64.5 mV	-74.0 mV
g_L	Leak conductance	10.0 nS		
Δ_T	Slope factor	2.0 mV		
V_{peak}	Spike detection threshold	0.0 mV		
a	Subthreshold adaptation	0.0 nS		
b	Spike-triggered adaptation	10.0 pA	100.0 pA	50.0 pA
τ_w	Adaptation time constant	500.0 ms	500.0 ms	150.0 ms
V_{th}	Spike initiation threshold	-50.0 mV		
I_e	Constant external input current	0.0 pA		
E_{ex}	Excitatory reversal potential	0.0 mV		
E_{in}	Inhibitory reversal potential	-80.0 mV		
V_m	Initialization of the voltage membrane	-64.5 mV	-64.5 mV	-47.5 mV
W	Initialization of adaptation current	0.0 pA		

B7	NEST : Inhibitory Neuron Model Parameters			
	case	Asynchronous	Irregular Synchronous	Regular bursting
C_m	Capacity of the membrane	200.0 pF		
t_{ref}	Duration of refractory period	5.0 ms		
V_{reset}	Reset value for V_m after a spike	-65.0 mV	-65.0 mV	-75.0 mV
E_L	Leak reversal potential	-65.0 mV	-65.0 mV	-75.0 mV
g_L	Leak conductance	10.0 nS		
Δ_T	Slope factor	0.5 ms		
V_{peak}	Spike detection threshold	0.0 mV		
a	Subthreshold adaptation	0.0 nS		
b	Spike-triggered adaptation	0.0 pA		
τ_w	Adaptation time constant	1.0 ms		
V_{th}	Spike initiation threshold	-50.0 mV		
I_e	Constant external input current	0.0 pA		
E_{ex}	Excitatory reversal potential	0.0 mV		
E_{in}	Inhibitory reversal potential	-80.0 mV		
V_m	Initialization of the voltage membrane	-65.0 mV	-65.0 mV	-75.0 mV
W	Initialization of adaptation current	0.0 pA		

B8	NEST : Connectivity between regions			
parameter synapses				
τ_{ex}	Rise time of excitatory synaptic conductance			5.0ms
τ_{in}	Rise time of inhibitory synaptic conductance			5.0ms
**Name**	**Source**	**Target**	**Weights**	**Pattern**
EE_global	E	E I	1.0	Fixed total number of connections from one to another region. The number of synapses to another region is A: 1150000, IS: 3000000 and RB: 800000. The delay (161.6 ms) is defined by the multiplication of velocity (3.0 mm/ms) and distance between regions (53.855 mm). See for more details in the section TVB: connectivity because delays and the weights are extracted from the connectivity of TVB.

B9	NEST : Connectivity inside the regions			
**Name**	**Source**	**Target**	**Weights**	**Pattern**
EE	E	E	1.0	Fixed number of input synapses ($N_e * p_{connect}$: A and SI $400 = 8000 * 0.05$ and RB $40 = 8000 * 0.005$). Neuron can connect to itself and can have multiple connections with another neuron.
EI	E	I	1.0	Fixed number of input synapses ($N_e * p_{connect}$: A and SI $400 = 8000 * 0.05$ and RB $40 = 8000 * 0.005$). Neuron can have multiple connections with another neuron.
IE	I	E	g	Fixed number of input synapses ($N_i * p_{connect}$: A and SI $100 = 2000 * 0.05$ and RB $10 = 2000 * 0.005$). Neuron can have multiple connections with another neuron. The weight equals 10.0 for A, 5.0 for SI and 10.0 for RB.
II	I	I	g	Fixed number of input synapses ($N_i * p_{connect}$: A and SI $100 = 2000 * 0.05$ and RB $10 = 2000 * 0.005$). Neuron can connect to itself and can have multiple connections with another neuron. The weight equals 10.0 for A, 5.0 for SI and 10.0 for RB.

B10	NEST : Input		
Poisson generator			
equation	$p(n) = \frac{\lambda^n}{n!} \exp(-\lambda)$		
implementation algorithm	Ahrens and Dieter 1982		
case	A	IS	RB
excitatory firing rate λ_{ex}	1.0	0.0	0.0
inhibitory firing rate λ_{in}	0.0	0.0	0.0
weight connection	1.0	1.0	1.0
Spike generator			
Proxy for the input of the region simulated with TVB. (see the section transformation TVB to NEST)			

B11	NEST : Measurement (part 1)		
state variable	Voltage membrane, adaptation current	precision	0.1
		number of recorded neurons	10 excitatory and 10 inhibitory
	spike time	precision	0.1 ms
		number of recorded neurons	all
spike activities	raster plot	precision	0.1 ms
	histogram of instantaneous firing rate	bins	0.1ms
	simple moving average	windows size	T (20ms)
	spectogram	method	Welch's method
		sampling frequencies	10^4Hz
		window shape	Hann window
		length of each segment	10^4
		length of the FFT	10^4
		number of points overlapping	5.10^3
		detrend	removing the mean
		sides	only real part

B11	NEST : Measurement (part 2)	
Micro -electrodes: Local Field Potential	software<br>number of MPI<br>number random seed	HybridLFPy[8]<br>2<br>2
	number of segment by neuron	defined by the method lambda100 of Neuron
	resolution	0.1 ms
	soma position	random in a cylinder of radius 2000 mm and height of 100mm with a minimal distance of 1mm. The centre of the cylinder is (0,-400).
	excitatory neurons	
	morphology	pyramidal cell of Shuman 2020 [9] without biophysics and synapses mechanisms
	initial membrane potential	V_m (-64.5 mV or -47.5 mV)
	axial resistance	150.0 Ohm
	membrane capacitance	C_m (200pF)
	passive mechanism	yes
	passive reversal potential	E_L (-64.5 mV or -74.0 mV))
	passive conductance	g_L (10 nS)
	inhibitory neurons	
	morphology	basket cell of Shuman 2020 [9] without biophysics and synapses mechanisms
	initial membrane potential	V_m (-64.5 mV or -75.0 mV)
	axial resistance	150.0 Ohm
	membrane capacitance	C_m (200pF)
	passive mechanism	yes
	passive reversal potential	E_L (-65.0 mV or -75.0 mV))
	passive conductance	g_L (10 nS)

<table>
<tr><th>B11</th><th colspan="2">NEST : Measurement(part 3)</th></tr>
<tr><td rowspan="20">Micro -electrodes: Local Field
Potential</td><td colspan="2">connectivity</td></tr>
<tr><td>layers</td><td>2 : [[300,-100],[-100,-600]]</td></tr>
<tr><td>synapse shape</td><td>truncated exponential</td></tr>
<tr><td>delay and weight distribution</td><td>homogeneous values by population</td></tr>
<tr><td>excitatory connection by layers and populations</td><td>$\begin{array}{c|c} N_e * p_{connect} * 0.5 & 0 \\ \hline N_e * p_{connect} * 0.5 & N_i * p_{connect} \end{array}$</td></tr>
<tr><td>excitatory weight</td><td>1.0</td></tr>
<tr><td>excitatory delay</td><td>dt (0.1 ms)</td></tr>
<tr><td>inhibitory connection by layers and populations</td><td>$\begin{array}{c|c} 0 & 0 \\ \hline N_e * p_{connect} & N_i * p_{connect} \end{array}$</td></tr>
<tr><td>inhibitory weight</td><td>g (5.0 or 10.0)</td></tr>
<tr><td>inhibitory delay</td><td>dt (0.1 ms)</td></tr>
<tr><td colspan="2">electrodes</td></tr>
<tr><td>extracellular conductivity</td><td>0.3 S</td></tr>
<tr><td>electrode positions and contacts surface normal</td><td><table>
<tr><th colspan="3">positions</th><th colspan="3">normal</th></tr>
<tr><th>x</th><th>y</th><th>z</th><th>x</th><th>y</th><th>z</th></tr>
<tr><td>1273</td><td>1273</td><td>1273</td><td>1</td><td>1</td><td>0</td></tr>
<tr><td>1273</td><td>-1273</td><td>-1273</td><td>1</td><td>1</td><td>0</td></tr>
<tr><td>-1273</td><td>-1273</td><td>15</td><td>1</td><td>1</td><td>0</td></tr>
<tr><td>-15</td><td>15</td><td>-15</td><td>1</td><td>1</td><td>0</td></tr>
<tr><td>1288</td><td>1258</td><td>1288</td><td>1</td><td>-1</td><td>0</td></tr>
<tr><td>1258</td><td>1288</td><td>1258</td><td>1</td><td>-1</td><td>0</td></tr>
<tr><td>1288</td><td>1258</td><td>-1800</td><td>1</td><td>-1</td><td>0</td></tr>
<tr><td>-1800</td><td>-1800</td><td>-1800</td><td>1</td><td>-1</td><td>0</td></tr>
<tr><td>-385</td><td>-385</td><td>-415</td><td>1</td><td>0</td><td>0</td></tr>
<tr><td>-415</td><td>-385</td><td>-385</td><td>1</td><td>0</td><td>0</td></tr>
<tr><td>-415</td><td>-415</td><td>-385</td><td>1</td><td>0</td><td>0</td></tr>
<tr><td>-385</td><td>-415</td><td>-415</td><td>1</td><td>0</td><td>0</td></tr>
</table></td></tr>
<tr><td>contact shape</td><td>circle of radius 20 mm</td></tr>
<tr><td>number of discrete point for compute the average potential</td><td>20</td></tr>
<tr><td>assumption method</td><td>soma as point</td></tr>
</table>

C1	TVB : Model Summary
Neural Mass model	Mean Adaptive Exponential
Connectivity	Mouse connectome with 104 regions
Coupling	linear coupling
stimulus	--
Monitors	ECoG (Electrocorticography) and state variable of the mean-field

C2	TVB : Coupling
Name	Linear
Type	Linear coupling
equations	$$\nu_{ext_k} = a * \left(\sum_{104}^{j=1} u_{kj} \nu_{e_j}(t - \tau_{kj}) \right) + b$$ where u_{kj} are the elements of the weights matrix, τ_{kj} are the elements of the delay matrix and ν_{e_j} are the mean excitatory firing rate of the regions j.
parameters	$a = 1.0$ and $b = 0.0$

C3	TVB : Connectivity
Connectivity is extracted from tracer data as explained by the paper TVBM[10]	
number of region	104
tract lengths	maximum : 115.46 and mean : 53.58
speed	3 ms
weights	The weights are normalized such as the sum of the input weight to one region equals 1. (maximum: 0.73 and mean: 0.02)
centers	average center of mouse brain : [57., 74.97, 42.53]
orientation	the orientation is defined by a vector from the average centre of a mouse brain to the centre of the regions

C3	TVB : Connectivity
region name	the region names are extracted from Allen Mouse Brain Connectivity Atlas (17/01/2017)[11]
	Right Primary motor area, Right Secondary motor area, Right Primary somatosensory area nose, Right Primary somatosensory area barrel field, Right Primary somatosensory area lower limb, Right Primary somatosensory area mouth, Right Primary somatosensory area upper limb, Right Supplemental somatosensory area, Right Gustatory areas, Right Visceral area, Right Dorsal auditory area, Right Primary auditory area, Right Ventral auditory area, Right Primary visual area, Right Anterior cingulate area dorsal part, Right Anterior cingulate area ventral part, Right Agranular insular area dorsal part, Right Retrosplenial area dorsal part, Right Retrosplenial area ventral part, Right Temporal association areas, Right Perirhinal area, Right Ectorhinal area, Right Main olfactory bulb, Right Anterior olfactory nucleus, Right Piriform area, Right Cortical amygdalar area posterior part, Right Field CA1, Right Field CA3, Right Dentate gyrus, Right Entorhinal area lateral part, Right Entorhinal area medial part dorsal zone, Right Subiculum, Right Caudoputamen*, Right Nucleus accumbens*, Right Olfactory tubercle*, Right Substantia innominata*, Right Lateral hypothalamic area*, Right Superior colliculus sensory related*, Right Inferior colliculus*, Right Midbrain reticular nucleus*, Right Superior colliculus motor related*, Right Periaqueductal gray*, Right Pontine reticular nucleus caudal part*, Right Pontine reticular nucleus*, Right Intermediate reticular nucleus*, Right Central lobule*, Right Culmen*, Right Simple lobule*, Right Ansiform lobule*, Right Paramedian lobule*, Right Copula pyramidis*, Right Paraflocculus*, Left Primary motor area, Left Secondary motor area, Left Primary somatosensory area nose, Left Primary somatosensory area barrel field, Left Primary somatosensory area lower limb, Left Primary somatosensory area mouth, Left Primary somatosensory area upper limb, Left Supplemental somatosensory area, Left Gustatory areas, Left Visceral area, Left Dorsal auditory area, Left Primary auditory area, Left Ventral auditory area, Left Primary visual area, Left Anterior cingulate area dorsal part, Left Anterior cingulate area ventral part, Left Agranular insular area dorsal part, Left Retrosplenial area dorsal part, Left Retrosplenial area ventral part, Left Temporal association areas, Left Perirhinal area, Left Ectorhinal area, Left Main olfactory bulb, Left Anterior olfactory nucleus, Left Piriform area, Left Cortical amygdalar area posterior part, Left Field CA1, Left Field CA3, Left Dentate gyrus, Left Entorhinal area lateral part, Left Entorhinal area medial part dorsal zone, Left Subiculum, Left Caudoputamen*, Left Nucleus accumbens*, Left Olfactory tubercle*, Left Substantia innominata*, Left Lateral hypothalamic area*, Left Superior colliculus sensory related*, Left Inferior colliculus*, Left Midbrain reticular nucleus*, Left Superior colliculus motor related*, Left Periaqueductal gray*, Left Pontine reticular nucleus caudal part*, Left Pontine reticular nucleus*, Left Intermediate reticular nucleus*, Left Central lobule*, Left Culmen*, Left Simple lobule*, Left Ansiform lobule*, Left Paramedian lobule*, Left Copula pyramidis*, Left Paraflocculus*
cortical region	all the region name ending by a ’*’ are not cortical regions

C4	TVB : Neural Mass Model (part 1)
Name	Mean Ad Ex[12]
Type	Neural mass model of a network of adaptive exponential integrate and fire excitatory and inhibitory neurons of second statistical order with adaptation
equation	$\begin{aligned} T\,\frac{\partial \nu_e}{\partial t} =& (\mathcal{F}_e - \nu_e) + \frac{1}{2}\,c_{ee}\,\frac{\partial^2 \mathcal{F}_e}{\partial \nu_e \partial \nu_e} \\ & + \frac{1}{2}\,c_{ei}\,\frac{\partial^2 \mathcal{F}_e}{\partial \nu_e \partial \nu_i} + \frac{1}{2}\,c_{ie}\,\frac{\partial^2 \mathcal{F}_e}{\partial \nu_i \partial \nu_e} + \frac{1}{2}\,c_{ii}\,\frac{\partial^2 \mathcal{F}_e}{\partial \nu_i \partial \nu_i} \\ T\,\frac{\partial \nu_i}{\partial t} =& (\mathcal{F}_i - \nu_i) + \frac{1}{2}\,c_{ee}\,\frac{\partial^2 \mathcal{F}_i}{\partial \nu_e \partial \nu_e} \\ & + \frac{1}{2}\,c_{ei}\,\frac{\partial^2 \mathcal{F}_i}{\partial \nu_e \partial \nu_i} + \frac{1}{2}\,c_{ie}\,\frac{\partial^2 \mathcal{F}_i}{\partial \nu_i \partial \nu_e} + \frac{1}{2}\,c_{ii}\,\frac{\partial^2 \mathcal{F}_i}{\partial \nu_i \partial \nu_i} \\ T\,\frac{\partial c_{ee}}{\partial t} =& (\mathcal{F}_e - \nu_e)\,(\mathcal{F}_e - \nu_e) + c_{ee}\frac{\partial \mathcal{F}_e}{\partial \nu_e} + c_{ee}\frac{\partial \mathcal{F}_e}{\partial \nu_e} + c_{ei}\frac{\partial \mathcal{F}_i}{\partial \nu_e} \\ & + c_{ie}\frac{\partial \mathcal{F}_i}{\partial \nu_e} - 2c_{ee} + \frac{\mathcal{F}_e\,(1/T - \mathcal{F}_e)}{N_e} \\ T\,\frac{\partial c_{ei}}{\partial t} =& (\mathcal{F}_e - \nu_e)\,(\mathcal{F}_i - \nu_i) + c_{ee}\frac{\partial \mathcal{F}_e}{\partial \nu_e} \\ & + c_{ei}\frac{\partial \mathcal{F}_e}{\partial \nu_i} + c_{ei}\frac{\partial \mathcal{F}_i}{\partial \nu_e} + c_{ii}\frac{\partial \mathcal{F}_i}{\partial \nu_i} - 2c_{ei} \\ T\,\frac{\partial c_{ie}}{\partial t} =& (\mathcal{F}_i - \nu_i)\,(\mathcal{F}_e - \nu_e) + c_{ie}\frac{\partial \mathcal{F}_e}{\partial \nu_i} \\ & + c_{ee}\frac{\partial \mathcal{F}_e}{\partial \nu_e} + c_{ii}\frac{\partial \mathcal{F}_i}{\partial \nu_i} + c_{ie}\frac{\partial \mathcal{F}_i}{\partial \nu_e} - 2c_{ie} \\ T\,\frac{\partial c_{ii}}{\partial t} =& (\mathcal{F}_i - \nu_i)\,(\mathcal{F}_i - \nu_i) + c_{ie}\frac{\partial \mathcal{F}_e}{\partial \nu_i} + c_{ei}\frac{\partial \mathcal{F}_e}{\partial \nu_i} + c_{ii}\frac{\partial \mathcal{F}_i}{\partial \nu_i} \\ & + c_{ii}\frac{\partial \mathcal{F}_i}{\partial \nu_i} - 2c_{ii} + \frac{\mathcal{F}_i\,(1/T - \mathcal{F}_i)}{N_i} \\ \tau_{W_e}\frac{\partial W_e}{\partial t} &= -W_e + b_e \nu_e + a_e(\mu_V(\nu_e, \nu_i, \nu_{ext}, W_e) - EL_e) \\ \tau_{W_i}\frac{\partial W_i}{\partial t} &= -W_i + b_i \nu_i + a_i(\mu_V(\nu_e, \nu_i, \nu_{ext}, W_i) - EL_i) \end{aligned}$

C4	TVB : Neural Mass Model (part 2)
noise equation	Ornstein-Uhlenbeck process : $\tau_{ou}\frac{dou_t}{dt} = (\mu - ou_t) + \sigma\, dW_t$ with W_t is a Wiener process
transfer function	$\mathcal{F}_e = \mathcal{F}((\nu_e + 1e - 6) + w_\sigma ou_t, \nu_{ext}, \nu_i, W_e)$ $\mathcal{F}_i = \mathcal{F}((\nu_e + 1e - 6) + w_\sigma ou_t, \nu_{ext}, \nu_i, W_i)$ $\mathcal{F} = \frac{1}{2\,\tau_V} \cdot Erfc(\frac{V^{eff}_{thre} - \mu_V}{\sqrt{2}\,\sigma_V})$ $V^{eff}_{thre}(\mu_V, \sigma_V, \tau_V^N = \tau_V \frac{g_L}{Cm}) = P_0' + \sum_{x \in \{\mu_V, \sigma_V, \tau_V^N\}} P_x \cdot \left(\frac{x - x^0}{\delta x^0}\right) + \sum_{x,y \in \{\mu_V, \sigma_V, \tau_V^N\}^2} P_{xy} \cdot \left(\frac{x - x^0}{\delta x^0}\right)\left(\frac{y - y^0}{\delta y^0}\right)$ $\mu_G(\nu_e, \nu_{ext}, \nu_i) = ((\nu_e K_e + \nu_{ext} K_{ext})\tau_e Q_e) + (\nu_i K_i \tau_i Q_i) + g_L$ $\mu_{V_s}(\nu_e, \nu_{ext}, \nu_i, w, \mu_G) = \frac{((\nu_e K_e + \nu_{ext} K_{ext})\tau_e Q_e) E_e}{\mu_G} + \frac{(\nu_i K_i \tau_i Q_i) E_i + g_L EL_s - w}{\mu_G}$ $\sigma_V(\mu_V, \mu_G) = \sqrt{\sum_{s \in \{e,i\}} K_s \nu_s \frac{\left(\frac{Q_s}{\mu_G}(E_s - \mu_V)\tau_s\right)^2}{2\frac{Cm}{\mu_G} + \tau_s}}$ $\tau_V(\mu_V, \mu_G) = \frac{\sum_{s \in \{e,i\}} K_s \nu_s \left(\frac{Q_s}{\mu_G}(E_s - \mu_V)\tau_s\right)^2}{\sum_{s \in \{ex,in\}} K_s \nu_s \frac{\left(\frac{Q_s}{\mu_G}(E_s - \mu_V)\tau_s\right)^2}{2\frac{Cm}{\mu_G} + \tau_s}}$

C5	TVB : Neural Mass Model Parameters(part 1)			
	case	Asynchronous	Irregular Synchronize	Regular bursting
T	time resolution of the mean field	20.0ms		
C_m	Capacity of the membrane	200.0 pF		
EL_e	Leak reversal potential excitatory(E_L)	-64.5 mV	-64.5 mV	-74.0 mV
EL_i	Leak reversal potential inhibitory(E_L)	-65.0 mV	-65.0 mV	-75.0 mV
g_L	Leak conductance	10.0 nS		
a_e	Subthreshold adaptation of excitatory neurons(a)	0.0 nS		
b_e	Spike-triggered adaptation of excitatory neurons(b)	10.0 pA	100.0 pA	50.0 pA
τ_{W_e}	Adaptation time constant of excitatory neurons(τ_w)	500.0 ms	500.0 ms	150.0 ms
a_i	Subthreshold adaptation of inhibitory neurons(a)	0.0 nS		
b_i	Spike-triggered adaptation inhibitory neurons(b)	0.0 pA		
τ_{W_i}	Adaptation time constant of inhibitory neurons(τ_w)	1.0 ms		
E_e	Excitatory reversal potential(E_{ex})	0.0 mV		
τ_e	Rise time of excitatory synaptic conductance(τ_{ex})	5.0 ms		
Q_e	excitatory quantal conductance	1.0 nS		
E_i	Inhibitory reversal potential(E_{in})	-80.0 mV		
τ_i	Rise time of inhibitory synaptic conductance(τ_{in})	5.0 ms		
Q_i	inhibitory quantal conductance	10.0 nS	5.0 nS	10.0 nS
$p_{connect}$	probability of connection	0.05	0.05	0.005
N_{tot}	Number of total neurons	10000		
p_i	percentage of inhibitory neurons	0.2		

C5	TVB : Neural Mass Model Parameters(part 2)			
N_e	Number of excitatory neurons	$N_{tot}(1-p_i)$ =8000		
N_i	Number of inhibitory neurons	$N_{tot}p_i$ =2000		
K_e	mean number of input excitatory synapses : $N_e p_{connect}$	400	400	40
K_i	mean number of input inhibitory synapses : $N_i p_{connect}$	100	100	10
$Kext_e$	number of external excitatory synapse	115	300	80

P_e	second order polynomial of the phenomenological threshold for excitatory neurons in mV	P_0	P_{μ_V}	P_{σ_V}	$P_{\tau_V^N}$
		-0.0498	0.00506	-0.025	0.0014
		$P_{\mu_V^2}$	$P_{\sigma_V^2}$	$P_{(\tau_V^N)^2}$	
		-0.00041	0.0105	-0.036	
		$P_{\mu_V\sigma_V}$	$P_{\mu_V\tau_V^N}$	$P_{\sigma_V\tau_V^N}$	
		0.0074	-0.0012	-0.0407	
P_i	second order polynomial of the phenomenological threshold for inhibitory neurons in mV	P_0	P_{μ_V}	P_{σ_V}	$P_{\tau_V^N}$
		-0.0514	0.004	-0.0083	0.0002
		$P_{\mu_V^2}$	$P_{\sigma_V^2}$	$P_{(\tau_V^N)^2}$	
		-0.0005	0.0014	-0.014	
		$P_{\mu_V\sigma_V}$	$P_{\mu_V\tau_V^N}$	$P_{\sigma_V\tau_V^N}$	
		0.0045	0.0028	-0.00153	

ν_{ext}	external input	see coupling section		
w_σ	weight of the noise	0.0002	0.0006	0.002
σ	variation of the noise	0.2		
μ	mean of the noise	0.0		
τ_{ou}	mean of the noise	20.0		
	initial condition (random between maximum and minimum)	$\mu_E(kHz):(0.,0.)$ $\mu_i(kHz):(0.,0.)$<br>$c_{ee}:(0.,0.)$ $c_{ei}:(0.,0.)$<br>$c_{ii}:(0.,0.)$<br>$W_e(pA):(0.,5.)$ $W_i(pA):(0.,0.)$		

C6	TVB : Monitor	
state variable	proxy node	node
	Only the mean firing rate of the excitatory population because it's the coupling variable and it's extracted from NEST simulation	mean firing rate of excitatory and inhibitory population, the variation of excitatory and inhibitory firing rate, the co-variation between excitatory and inhibitory firing rate, mean adaptive current of excitatory and inhibitory firing rate
	precision	dt (0.1ms)
SEEG	equation	$\Psi_{ECoG}(channel, t) = P.\nu_e + noise$ where P is the gain matrix and N is the mean firing rate of excitatory population $P_{ij} = scaling\_factor * region\_volume_j/\|\|r_i - r_j\|\|$ where r_i is the position of the contact point of the channel and r_j is the centre of region j.
	contact position	see table below
	$scaling\_factor$	1.0
	$region\_volume$	The volume is extracted from the volume mapping of Allen Mouse Brain Connectivity Atlas<br>mean: 3712.29 max: 16245.0 min: 957.0

Contact position:

	x	y	z
left hemisphere	40.0	80.0	79.5
	20.0	80.0	72.0
	30.0	70.0	76.5
	30.0	90.0	75.5
	22.5	72.5	73.0
	22.5	87.5	72.5
	37.5	72.5	78.5
	37.5	87.5	78.5
right hemisphere	94.0	80.0	69.
	74.0	80.0	78.5
	84.0	70.0	74.5
	84.0	90.0	74.0
	76.5	72.5	77.5
	76.5	87.5	77.5
	91.5	72.5	70.5
	91.5	87.5	70.

D	Transformation NEST to TVB : model
Name	SMFR : sliding mean firing rate
Type	sliding mean over the histogram of the instantaneous firing rate
Input	spike trains of excitatory neurons from one brain region for synchronized time (2ms)
Output	mean firing rate of the excitatory population of a brain region for synchronized time (2ms)
equation	$\forall t \geq T, \text{SMFR}(t) = \frac{\sum_{s=t-T}^{t} \sum_{n=1}^{N_e} spike(n,s)}{N_e T} * 10^3$ (KHz)<br>where $spike(n,s) = \begin{cases} 1 \text{ if neuron n create spike at time s} \\ \quad \text{with a presicion of dt} \\ 0 \text{ else} \end{cases}$
parameters	size of the windows T : 20.0 ms (same as TVB)
parameters	number of neurons N_e : 8000 (same as NEST)
parameters	precision of the integration dt : 0.1 ms (same as TVB and NEST)
initialization	The transfer module doesn't have initialized because TVB used its initialization for starting the communication.

E	Transformation TVB to NEST : model	
	Name	MIP
	Type	Multiple Interaction Process[13]
	Input	incoming excitatory firing rate of a brain region for synchronized time (2ms)
	Output	spike trains to individual neurons with a correlation of p for synchronized time (2ms)
	equation	reference spike train:<br>$x_{ref}(t) = InhomogenousPoissonProcess((\nu_{input}(t)nb_{synapse}+1e-12)/p)$<br>input individual spike train to the neuron n:<br>$x_n(t) = x_{ref}B(size(x_{ref}), p)$
where	ν_{input}	mean external excitatory firing rate computed by TVB for the NEST population.
	$nb_{synapse}$	number of external input synapse (A:115, IS:300, RB:80)
	p	percentage of shared neurons (A:0.01, IS:0.1, RB:0.01)
	B	binomial law
	The implementation of the Inhomogenous Poisson Process	Dedicated function from the python library elephant (version 0.9), which: 1) generates spike trains with homogeneous Poisson generator for the highest rate; 2) removes some spikes for having variation of rate based on the input rates. The homogeneous Poisson generator computes the time interval between each spike using the exponential random generator of numpy.
	initialization	The initial rate sent to TVB are zeros during the first t_{synch} (2ms).

2.2 Figures

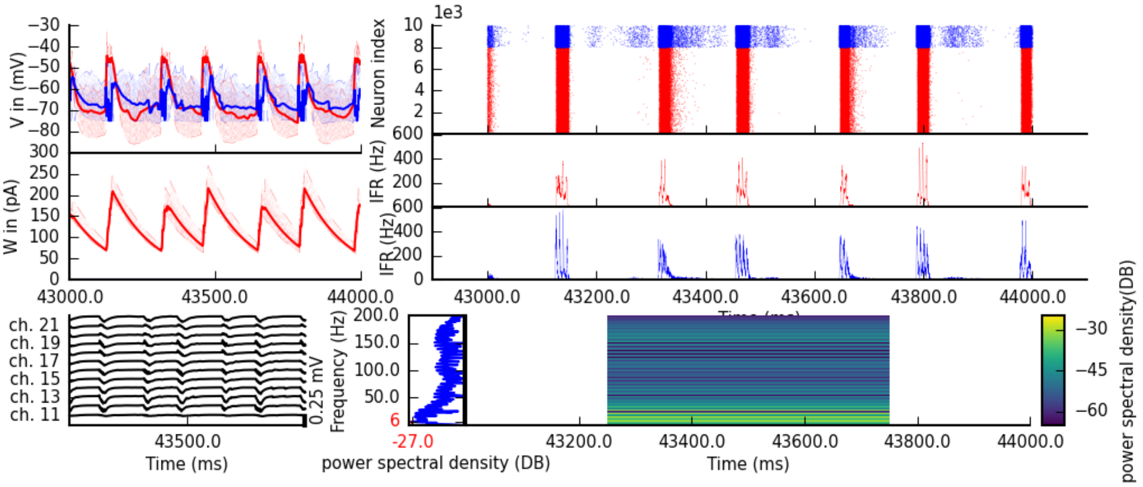

Figure 1. Zoom on 1s for the spiking neural network with regular bursting state
This figure is a zoom of the figure 3 between 43s and 44s. **top-left** Example of time series from 10 adaptive exponential leaky and integrator neurons. The red lines are excitatory and the blue curve are inhibitory neurons. The mean excitatory time series is shown with a thick red line and the inhibitory time series is shown with a thick blue line. **middle-left** The adaptation currents of 10 neurons are shown. The thick line is the mean adaptive currents. **bottom-left** The figure shows local field potential from the 12 sites of the middle line of the polytrode. The local field potential is computed from the spike trains of all neurons by the software HybridLFPY [8]. **top-right** The figure shows spike trains of 10000 neurons for 1s. **bottom-right** The figure shows respectively the excitatory and inhibitory instantaneous firing rate of the population in panel middle-right in red and blue. **bottom** Spectrogram and power spectrum example of the instantaneous firing rate for 1s.

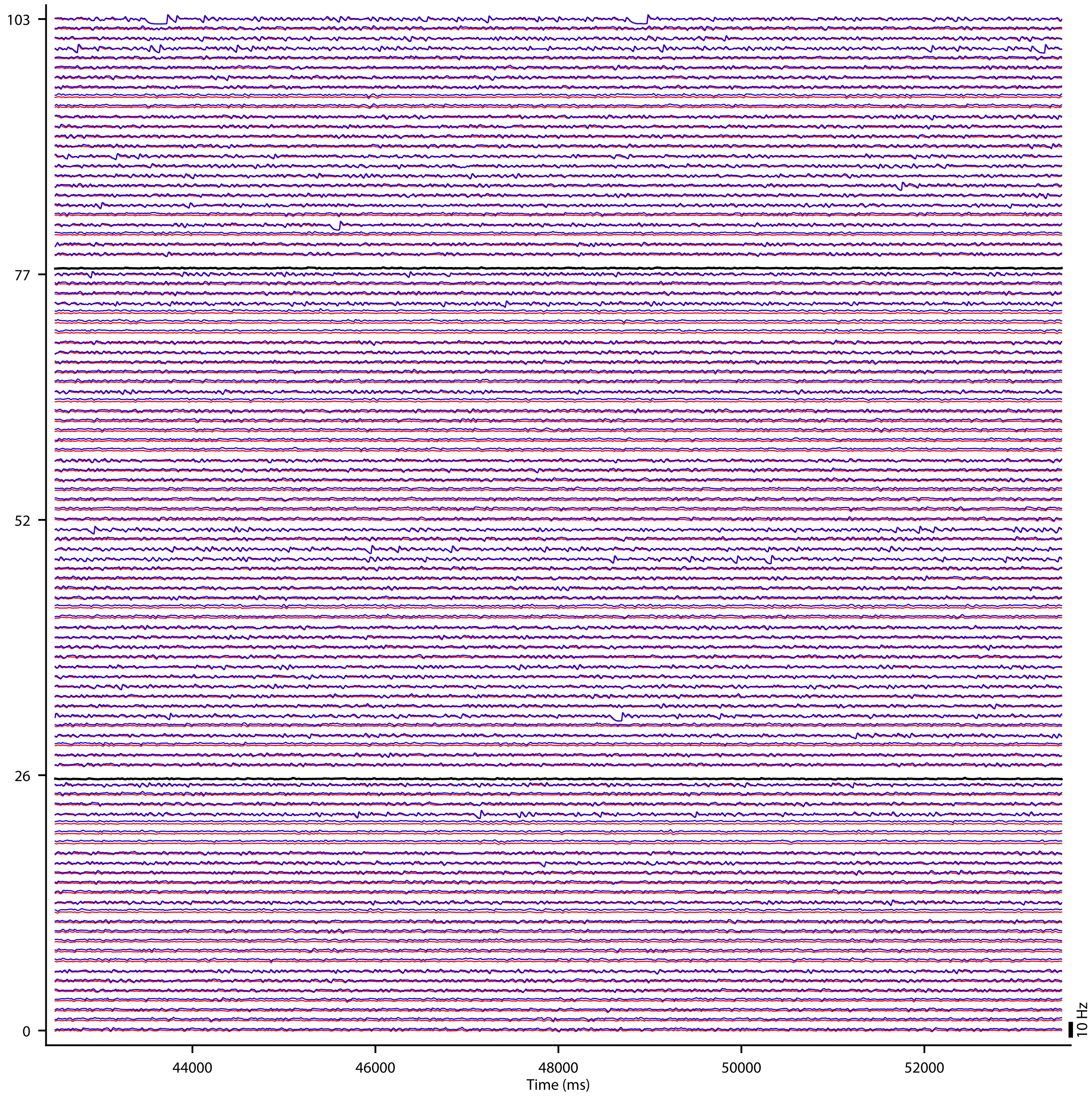

Figure 2. Mouse Brain activity for Asynchronous Irregular
For the state of the Asynchronous Irregular, an overview of the mean firing rates of excitatory, in red, and inhibitory, in blue, populations from the model of Mean Adaptive Exponential for all mouse brain regions. The two black curves are the mean firing rate of the two populations of excitatory neurons simulated with NEST [1].

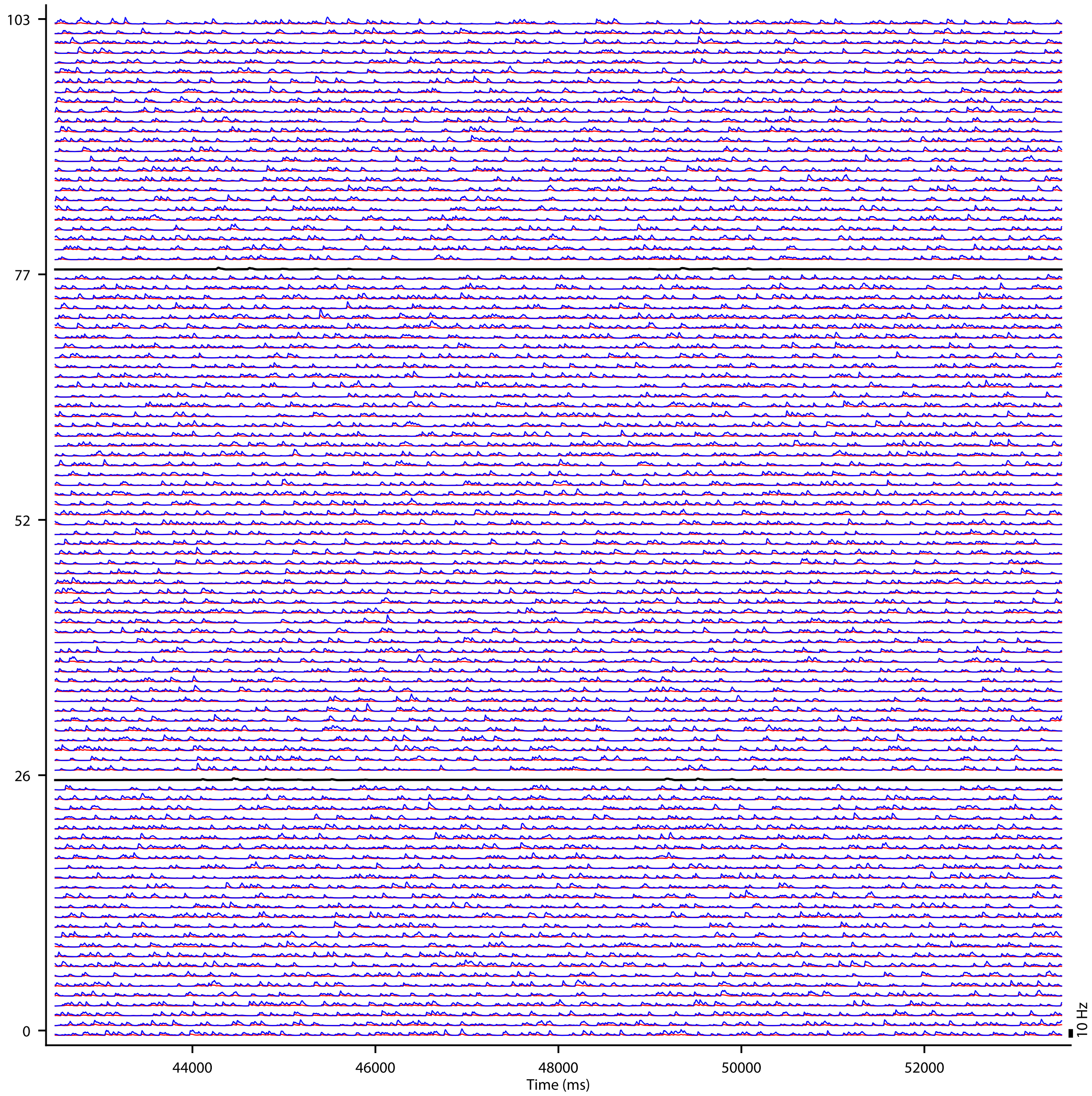

Figure 3. Mouse Brain activity for Synchronize Irregular
For the state of the Synchronize Irregular, an overview of the mean firing rates of excitatory, in red, and inhibitory, in blue, populations from the model of Mean Adaptive Exponential for all mouse brain regions. The two black curves are the mean firing rate of the two populations of excitatory neurons simulated with NEST [1].

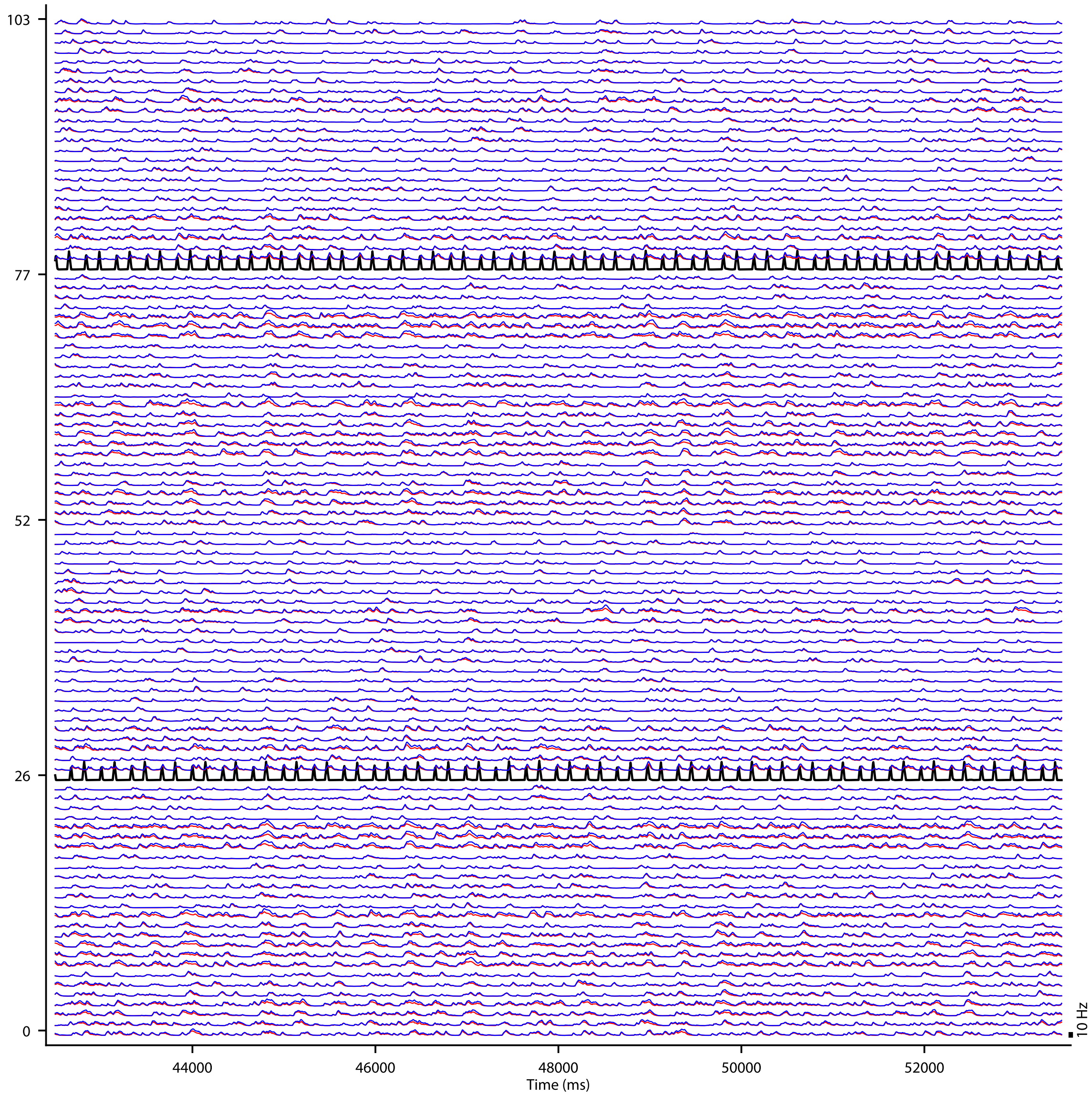

Figure 4. Mouse Brain activity for Regular Bursting
For the state of the Regular Bursting, an overview of the mean firing rates of excitatory, in red, and inhibitory, in blue, populations from the model of Mean Adaptive Exponential for all mouse brain regions. The two black curves are the mean firing rate of the two populations of excitatory neurons simulated with NEST [1].

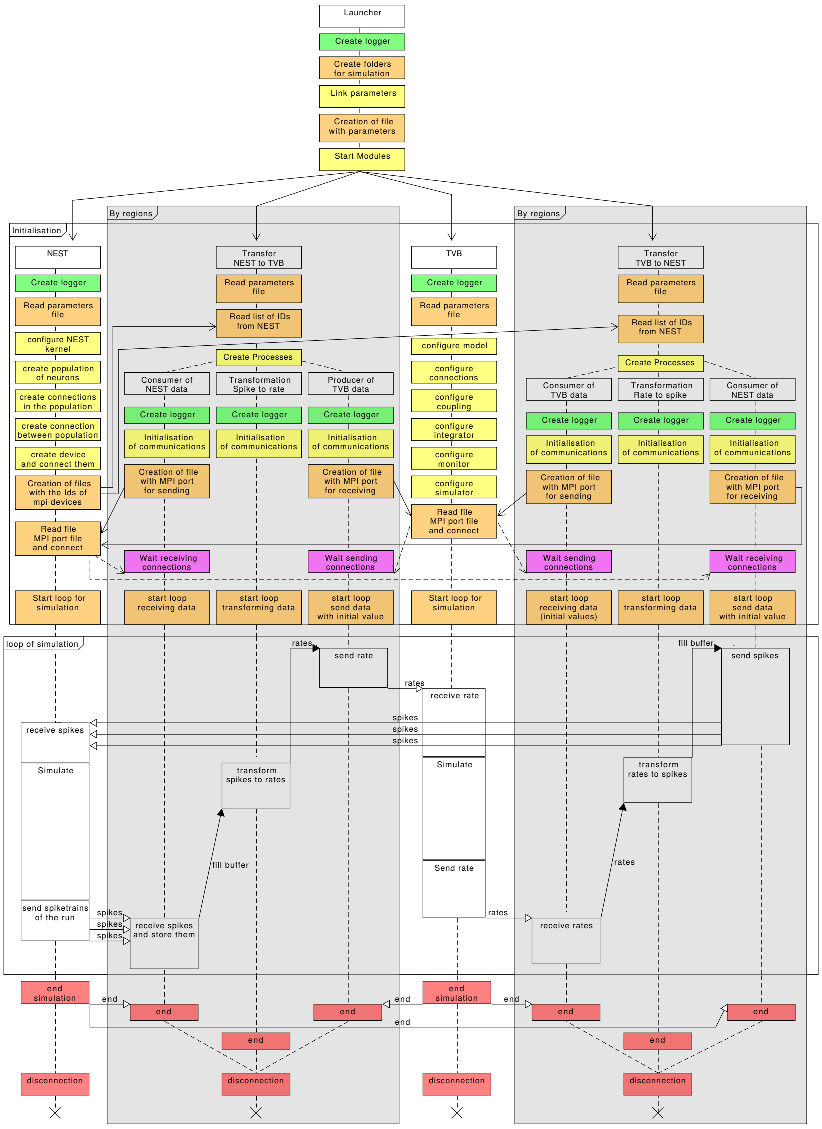

Figure 5. Detail sequence diagram of the co-simulation
This figure represents the interaction among the different modules during the co-simulation and the different exchanged data. The co-simulation is separated in 3 steps: initialisation and configuration, simulation and termination.
The colour code of the boxes :

- green for the creation of a logger, one by components and modules
- orange for access to file systems (the creation of a folder or a file, the reading of files, ...) and the start of the simulation with initial condition.
- yellow for initialization and configuration of modules and components
- magenta for MPI waiting connections
- white for the simulation step and the name of modules or components
- red for the termination of the simulation.

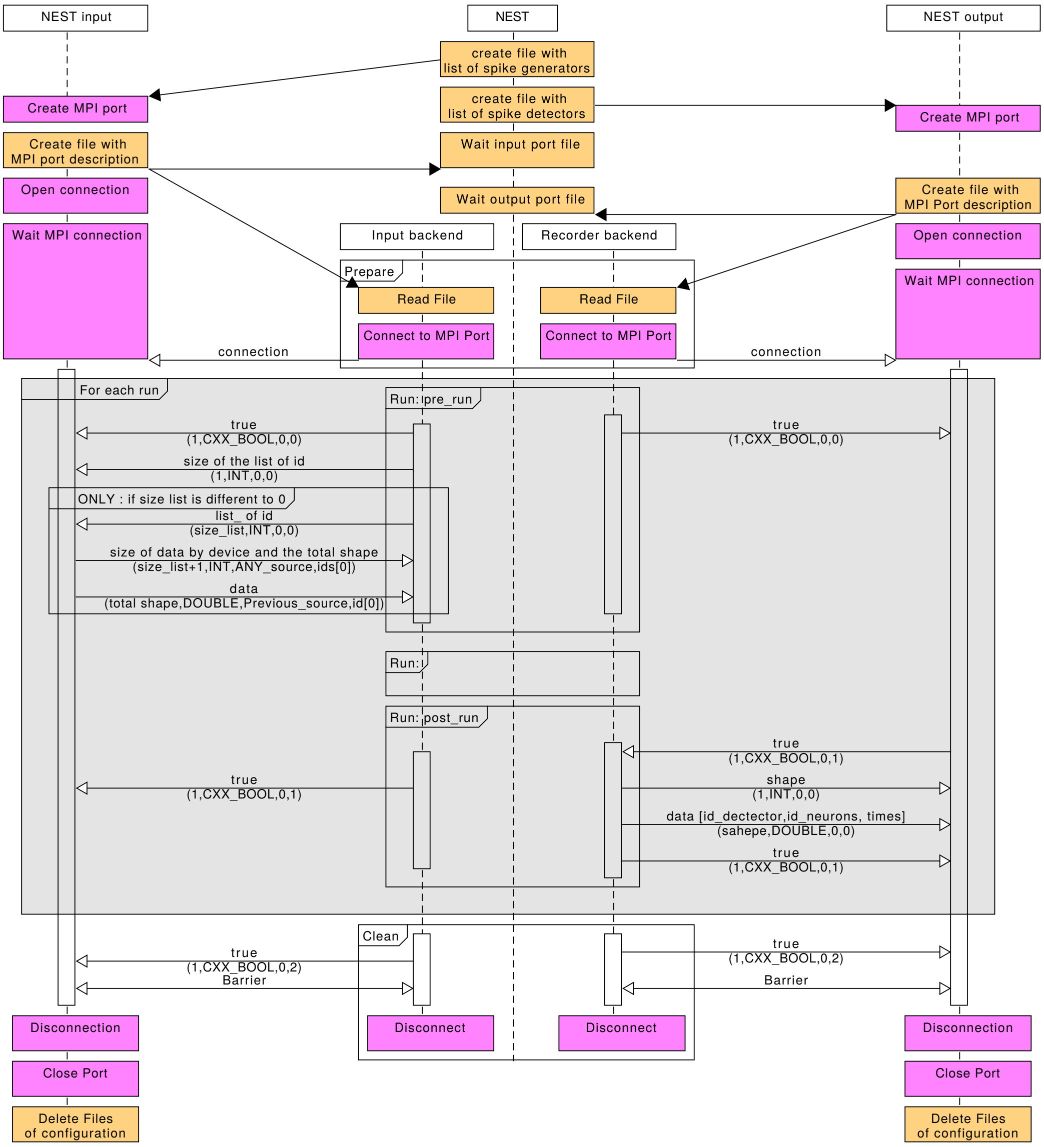

Figure 6. Sequence diagram of the communication protocol with NEST
The communication with NEST [1] is separated into 3 steps: creation of the MPI connection, simulation and termination.
The colour code of the boxes :

- orange for access to file systems (the creation of files, the reading of files, ...).
- magenta for management of MPI port
- white for the name of the modules or components

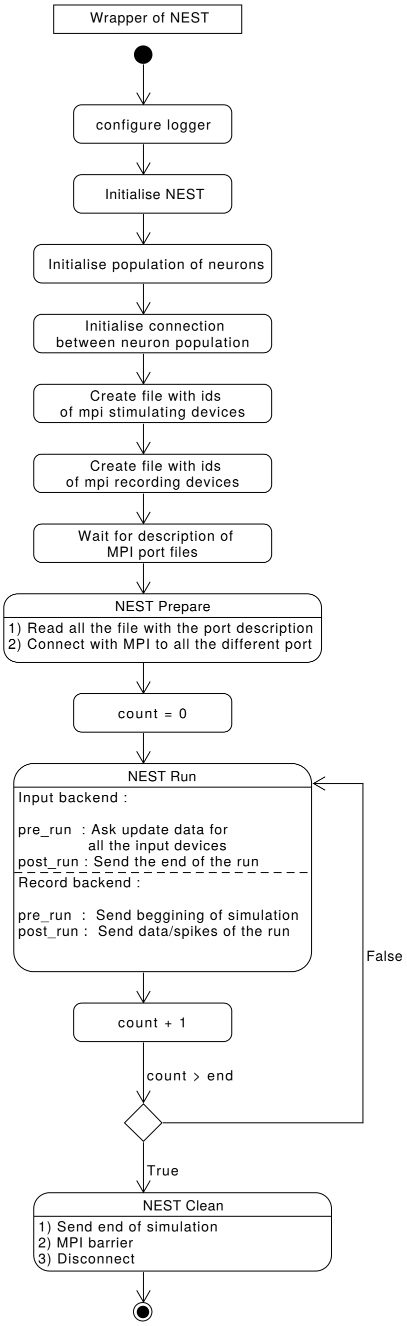

Figure 7. State diagram of NEST wrapper
The diagram describes all the different states of the NEST wrapper during the co-simulation. The beginning is the set-up of the network (the creation of neurons, their connection and the creation of devices). The additional steps are the loop of simulation and the termination.

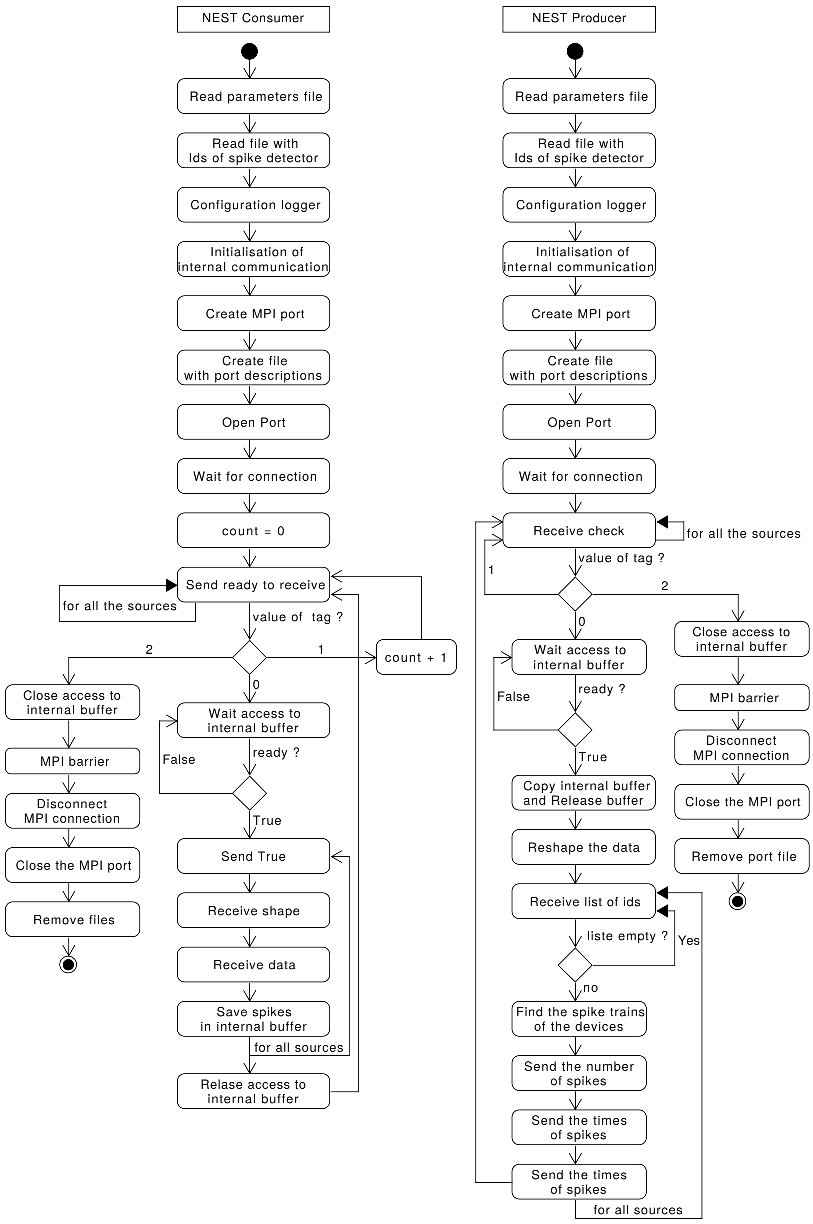

Figure 8. State diagram of transfer components for interaction with NEST
The diagram describes all the states of the components of the transfer module which communicates with NEST. The beginning is the configuration of itself and the creation of the MPI connection. Once the MPI connection is made, there is a loop of the simulation. The centre of the simulation loop is the value of the tag received by the component to identify if NEST is ready to receive or send messages. If this tag equals 2, the components go in the sequence for the termination phase.

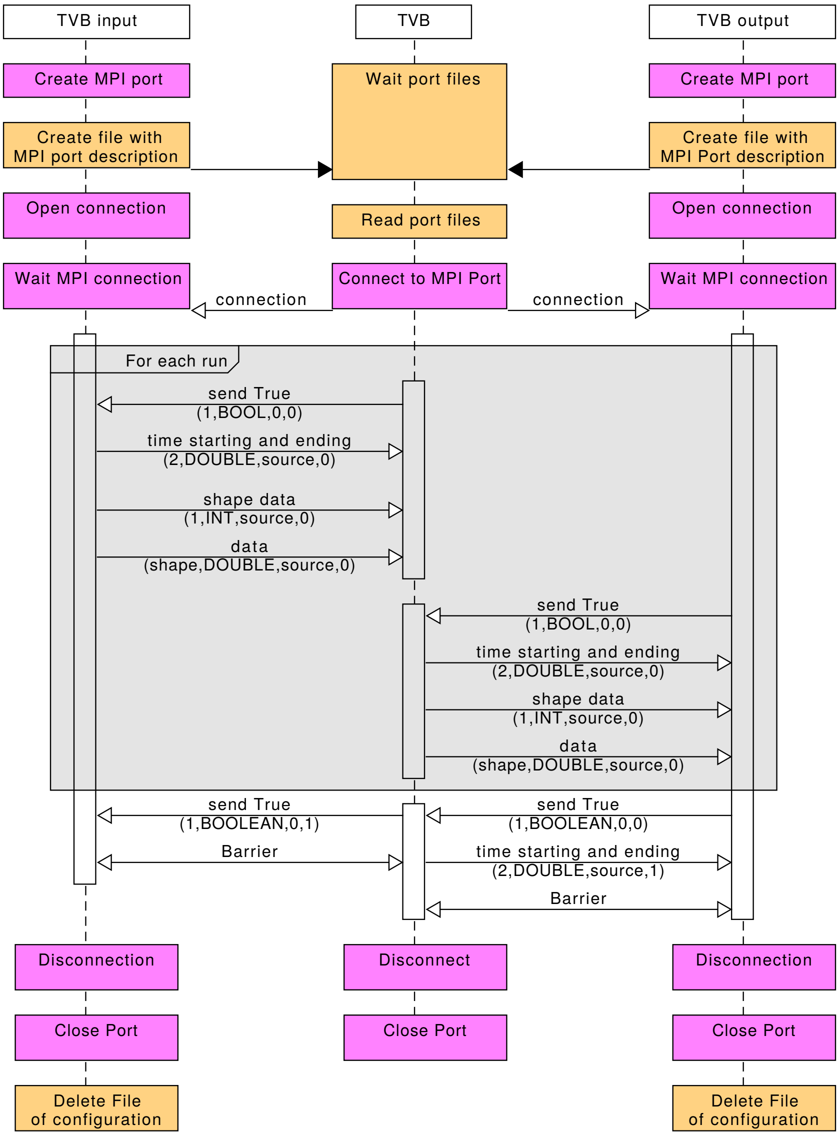

Figure 9. Sequence diagram of the communication protocol with the wrapper of TVB. The communication with TVB [6] is separated in 3 steps: creation of the MPI connection, simulation and termination.
The colour code of the boxes :

- orange for access to file systems (create files, read files, ...).
- magenta for management of MPI port
- white for the name of the modules or components

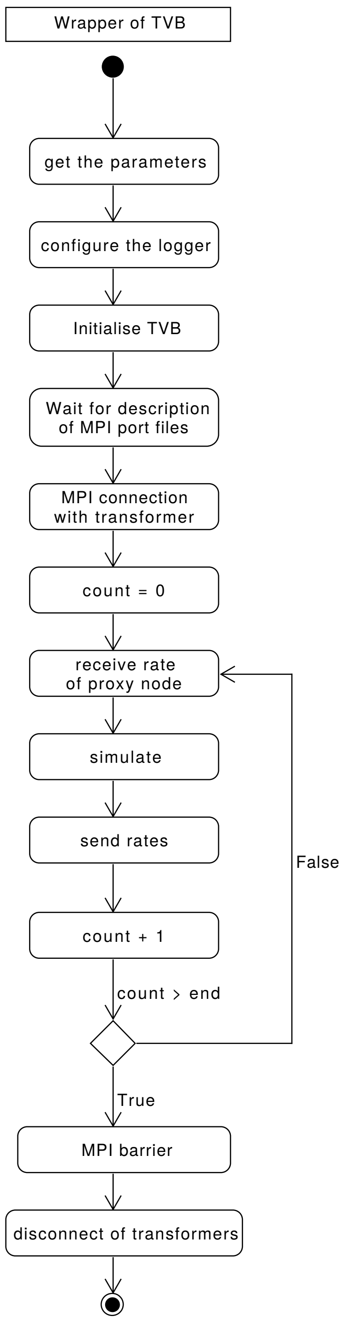

Figure 10. State diagram of wrapper of TVB modules
The diagram describes all the different states of the TVB wrapper during the co-simulation. The beginning is the set-up of the network (the creation of neurons, their connection and the creation of devices). The additional steps are the loop of simulation and the termination.

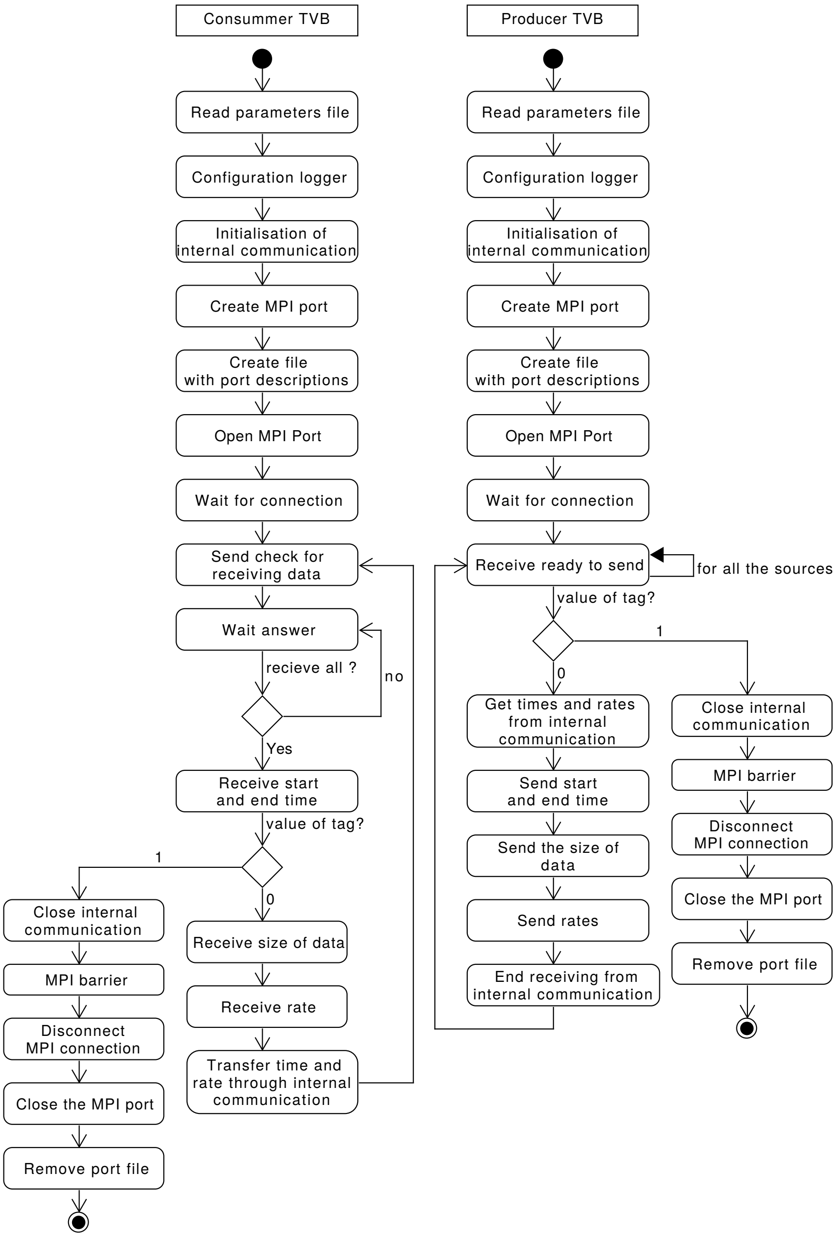

Figure 11. State diagram of transfer components for interaction with the wrapper of TVB The diagram describes all the state of the components of the transfer module which communicates with TVB. The beginning is the configuration of itself and the creation of the MPI connection. Once the MPI connection is made, there is a loop of the simulation. The centre of the simulation loop is the value of the tag received by the component to identify if NEST is ready to receive or send messages. If this tag equals 1, the components go in the sequence for the termination phase.

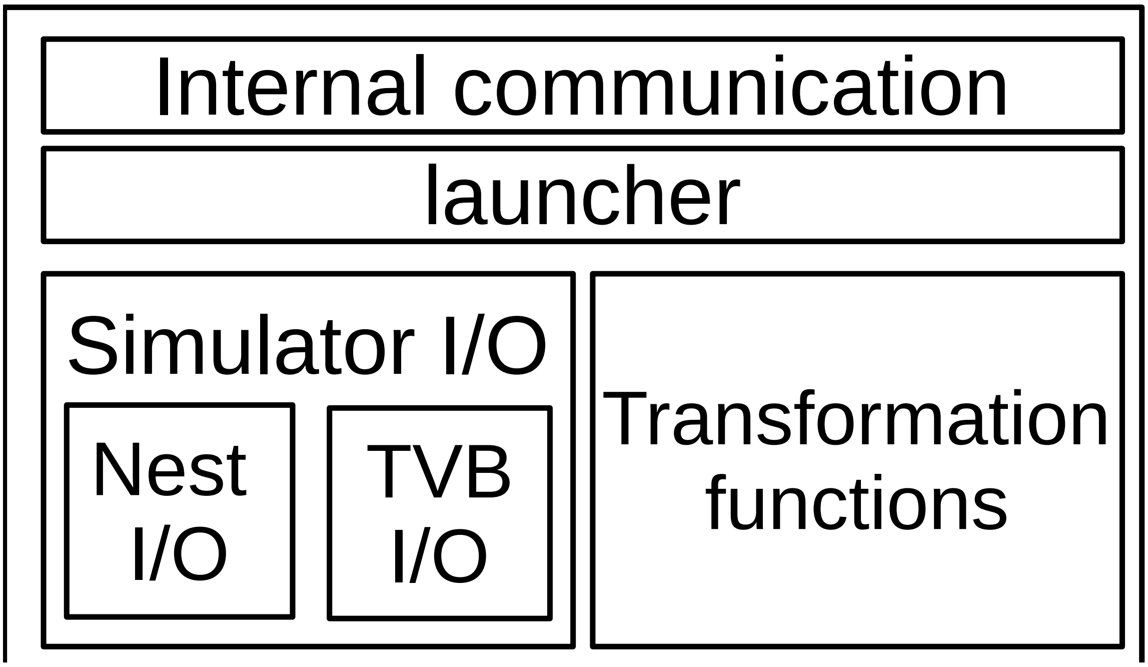

Figure 12. File organisation of the transformer module
The files of transfer modules are organised following the modularity of the module. The folder internal communication contains all the functions of the communication between components of the modules. The launcher regroups the files used to start the modules of transformation between specific simulators. The Simulator I/O contains the function for the interface of each simulator. The figure shows that the interface for each simulator (TVB and NEST) is separated and independent. Transformation functions are in a folder which contains the abstract class for the transformations and the implementation of specific transformations.

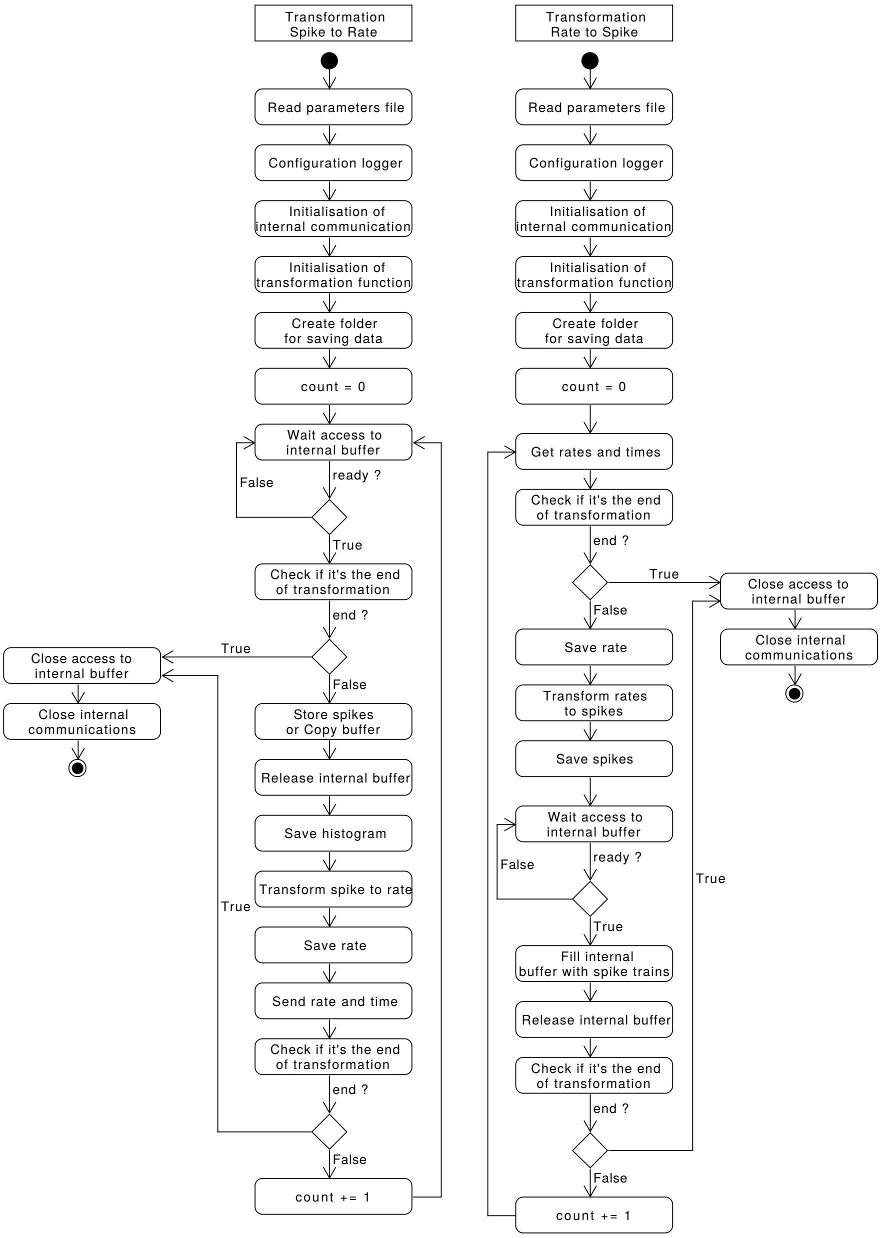

Figure 13. State diagram of transfer components which transform data from one scale to another
The diagram describes all the state of the transfer components. The beginning is the configuration of itself. The component is waiting to access to the data from one buffer for the transformation. After accessing to the data, it transforms them and awaits the access for writing in a second buffer. When it receives the termination from one side or the other, it goes in the sequence of termination.

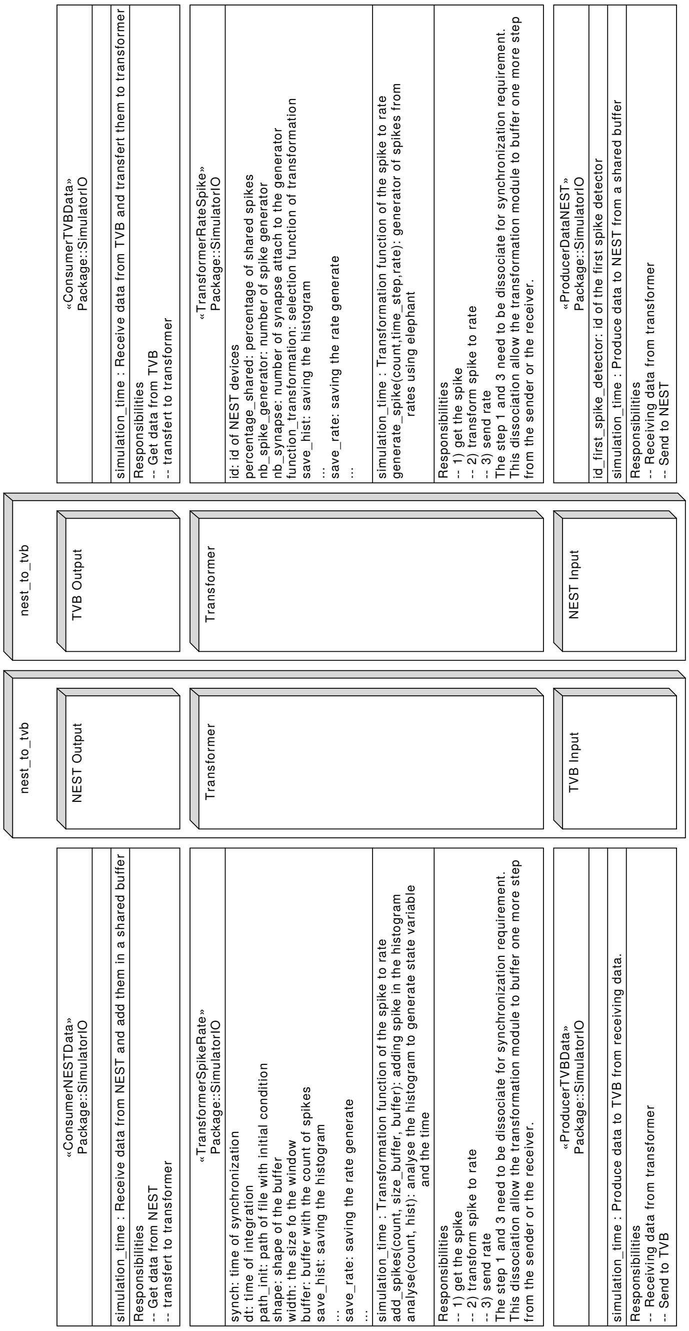

Figure 14. Structure diagram of the two transfer modules with the description of each component
Each component is based on a class. The diagram gives a short description of each class and their contents.

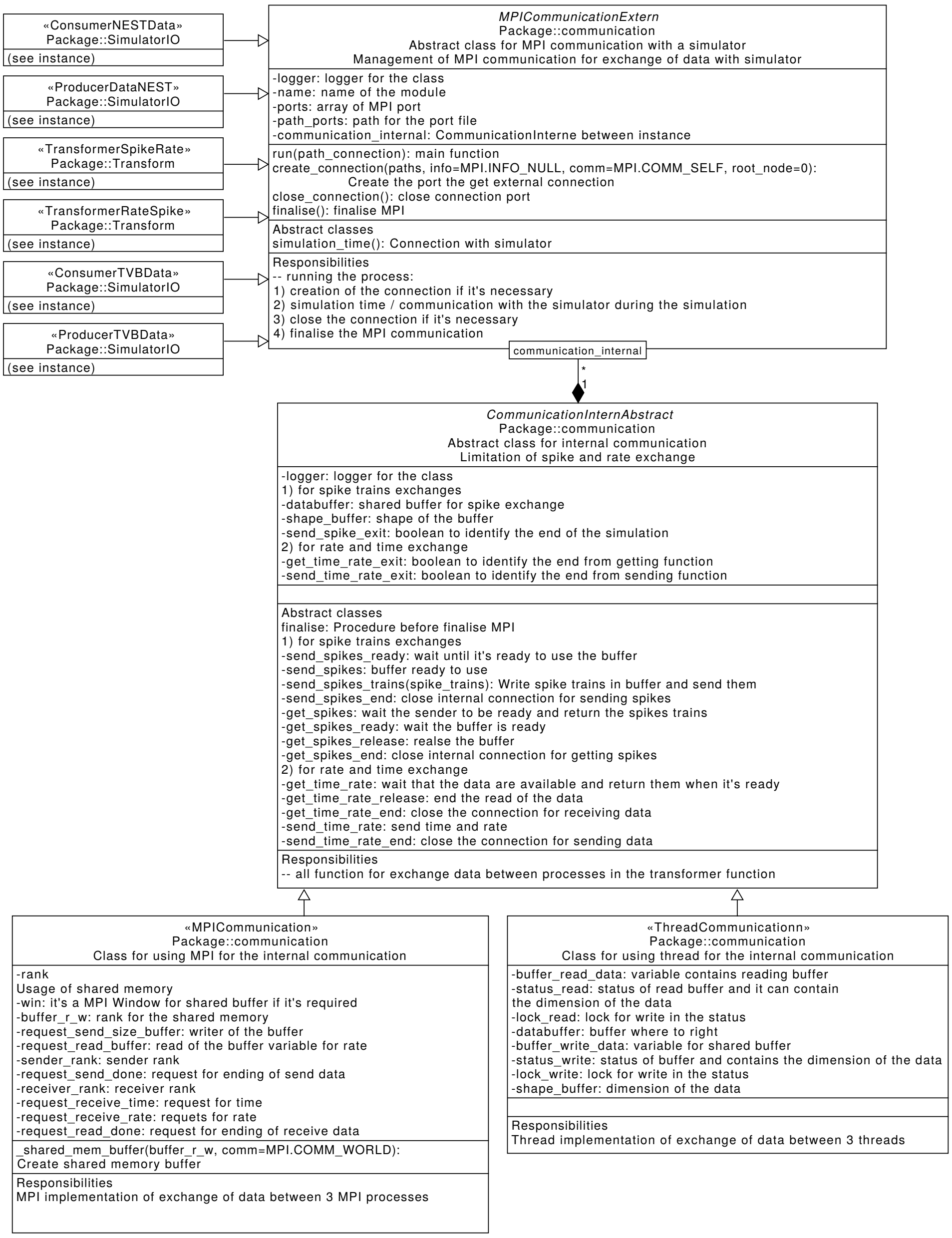

Figure 15. Class diagram of the transfer modules.
The description of the classes on the right part is described in the supplementary figure 14. All these classes inherit from an abstract class which manages MPI communication. This abstract class manage the MPI connection and has an internal communicator. This internal communicator is an abstract class for the communication between the transfer components. This internal communication can be a melting process or multithreading as the diagram shows.

a

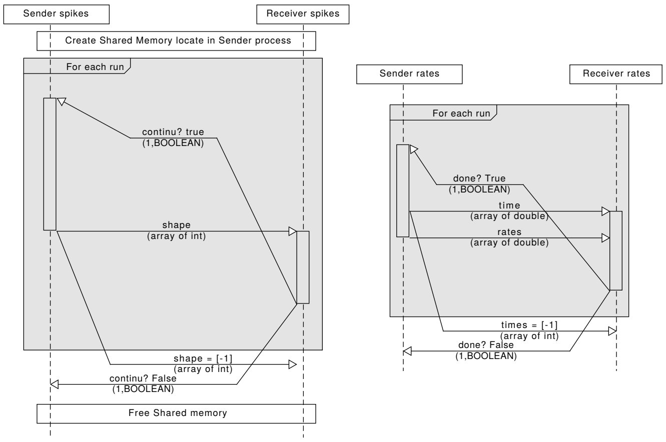

b

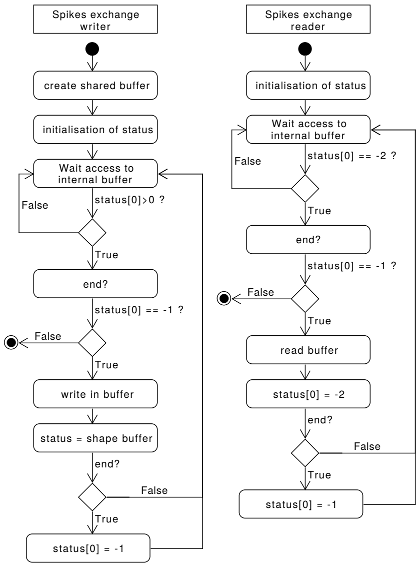

Figure 16. Communication between components in the transfer module
As described in the supplementary figure 15, the internal communication has 2 implementations. Panel **a** is a sequence diagram of the communication of spikes and rates using MPI communication. Panel **b** is a state diagram of the management of a shared buffer in the case of multithreading communication for transferring spike data.

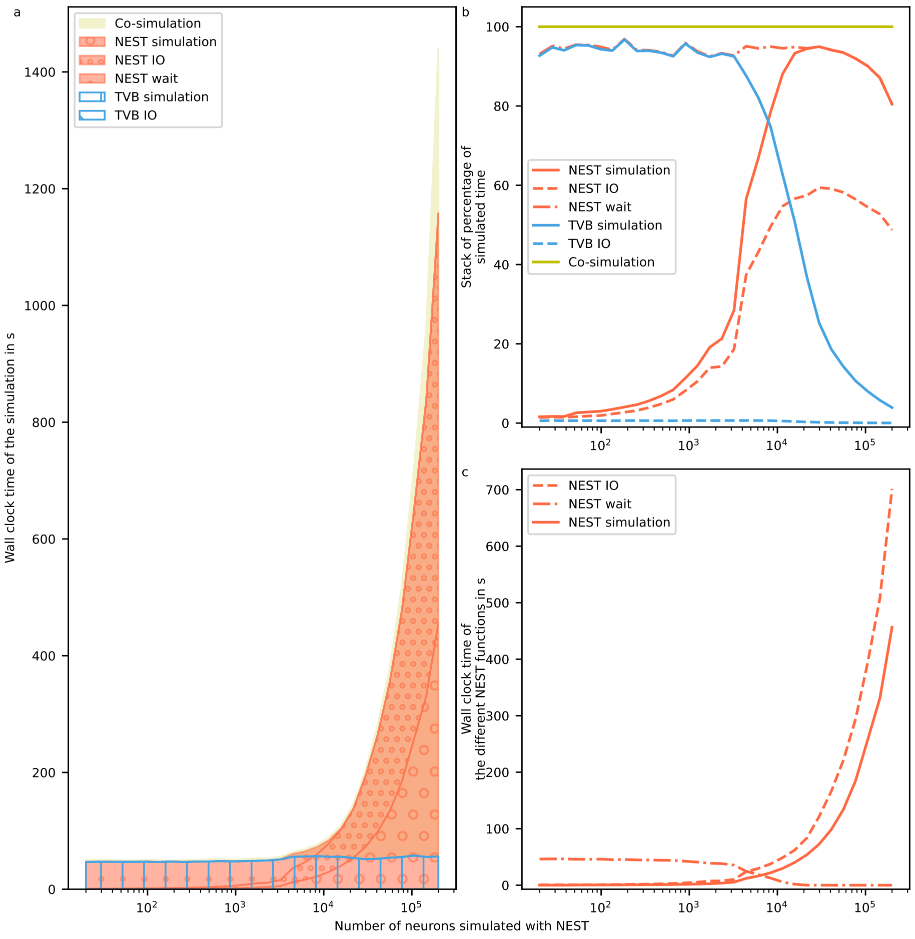

Figure 17. Details of the performance with the increase of neurons
Performance is obtained for 1 second of simulated time on a computer (see Materials and Methods for more details). The reference implementation uses 1 MPI process, 6 virtual processes/threads, and 2.0 ms to synchronize time between simulators for the simulation of 20000 neurons. Simulation time depends on the number of neurons simulated with NEST. **a** The wall clock time of the simulator depends on the number of neurons. The total time of the co-simulation is represented in yellow. The "simulation", "IO" and "wait" times of NEST are represented in red surface with respectively hatches of big circles, small circles and points. The "simulation" and "IO" times of TVB are represented in the blue surface with respective hatches of horizontal lines and oblique lines. **b** The wall clock time for the co-simulation (yellow curve), NEST (red curves) and TVB (blue curves) by the total wall clock time. The solid, dashed and dashed-dotted curves are associated with "simulation", "IO" and "wait" time of NEST. The solid and dashed line is associated with "simulation" and "IO" time of TVB. **c** The different timer for NEST simulator. Each contribution is reported as a red curve and for increasing numbers of neurons. The solid, dashed and dashed-dotted curves represent "simulation", "IO" and "wait" time of NEST, respectively.

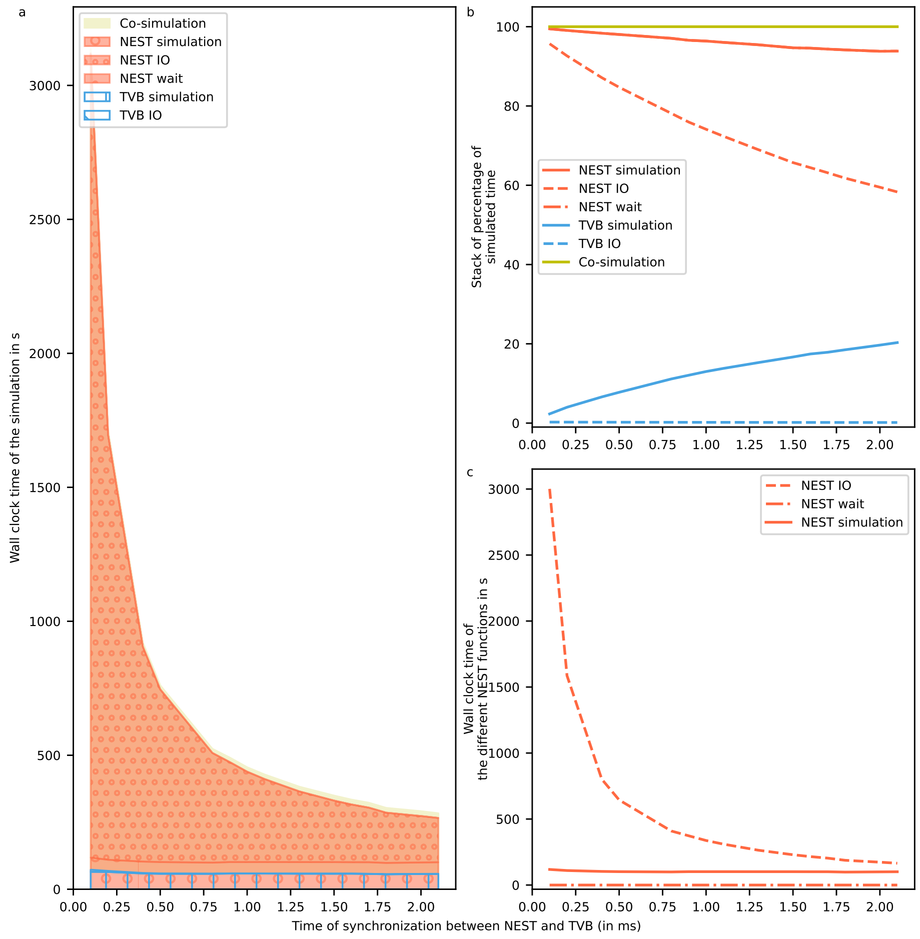

Figure 18. Details of the performance with the increase of synchronize time Performance is obtained for 1 second of simulated time on a computer (see Materials and Methods for more details). The reference implementation use 1 MPI process, 6 virtual processes/threads, 2.0 ms to synchronize time between simulator for the simulation of 20000 neurons. Simulation time depends on the synchronized time between NEST and TVB. **a** The wall clock time of the simulator depends on the time of synchronization between the two simulators. The total time of the co-simulation is represented in yellow. The ”simulation”, ”IO” and ”wait” times of NEST are represented in red surface with respectively hatches of big circles, small circles and points. The ”simulation” and ”IO” times of TVB are represented in the blue surface with respective hatches of horizontal lines and oblique lines. **b** The wall clock time for the co-simulation (yellow curve), NEST (red curves) and TVB (blue curves) by the total wall clock time. The solid, dashed and dashed-dotted curves are associated with ”simulation”, ”IO” and ”wait” time of NEST. The solid and dashed line is associated with ”simulation” and ”IO” time of TVB. **c** The different timer for NEST simulator. Each contribution is reported as red curves in function of number of neurons. The solid, dashed and dashed-dotted curves represent ”simulation”, ”IO” and ”wait” time of NEST, respectively.

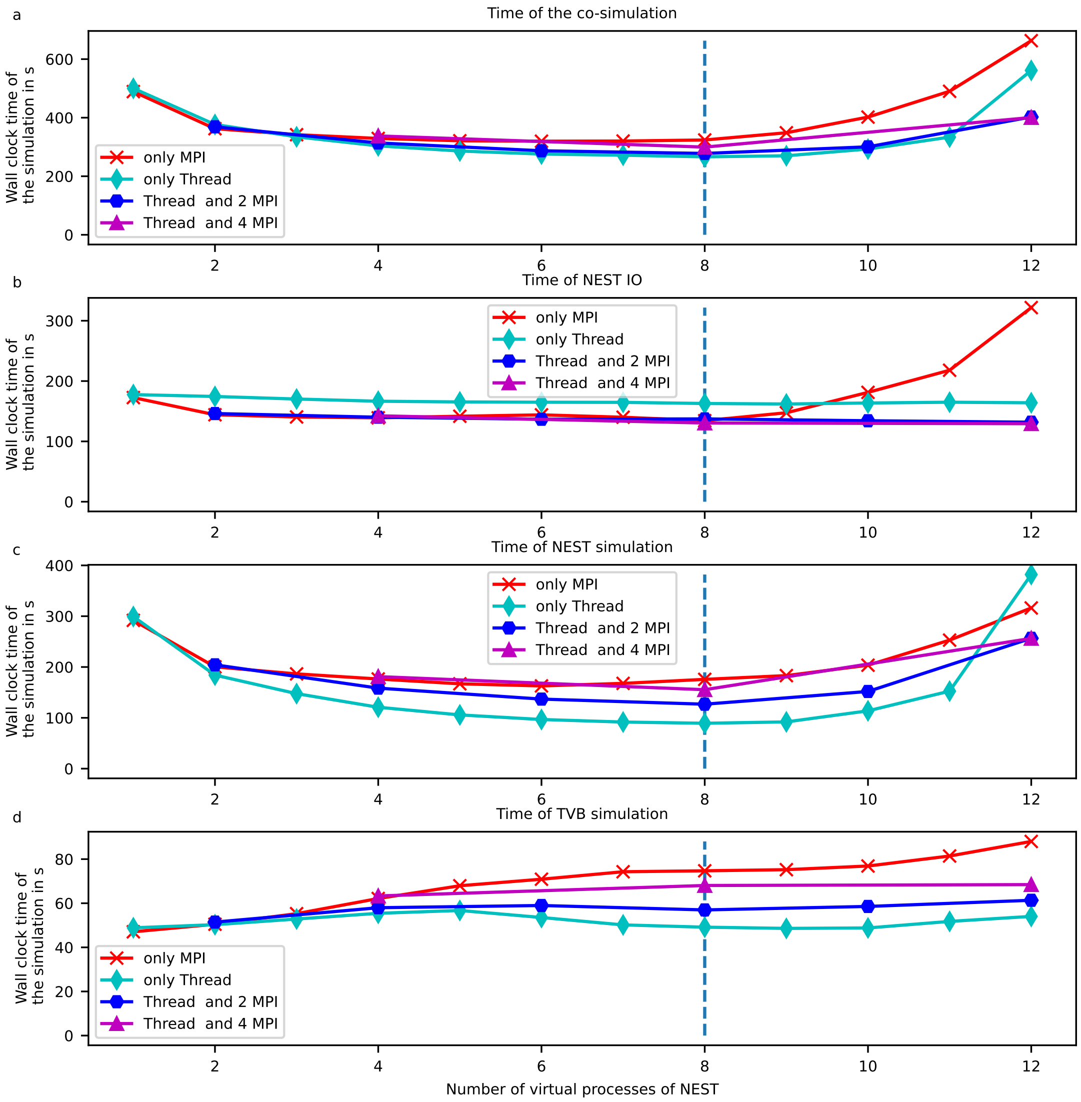

Figure 19. Details of the performance depending on the number of processes and threads for NEST
Performance is obtained for 1 second of simulated time on a computer (see Materials and Methods for more details). The reference implementation uses 1 MPI process, 6 virtual processes/threads, 2.0 ms to synchronize time between simulators for the simulation of 20000 neurons. Simulation time depends on the number of virtual processes used by NEST. The cyan, blue, purple, and red curves are associated with different parallelization strategies of NEST, respectively, only multithreading, 2 MPI processes with threads, 4 MPI processes with thread and only MPI processes. The horizontal blue line represents the number of cores of the computer **a** The total time of the co-simulation. **b** The ”IO” time of NEST **c** The ”simulation” time of NEST **d** The ”simulation” time of TVB

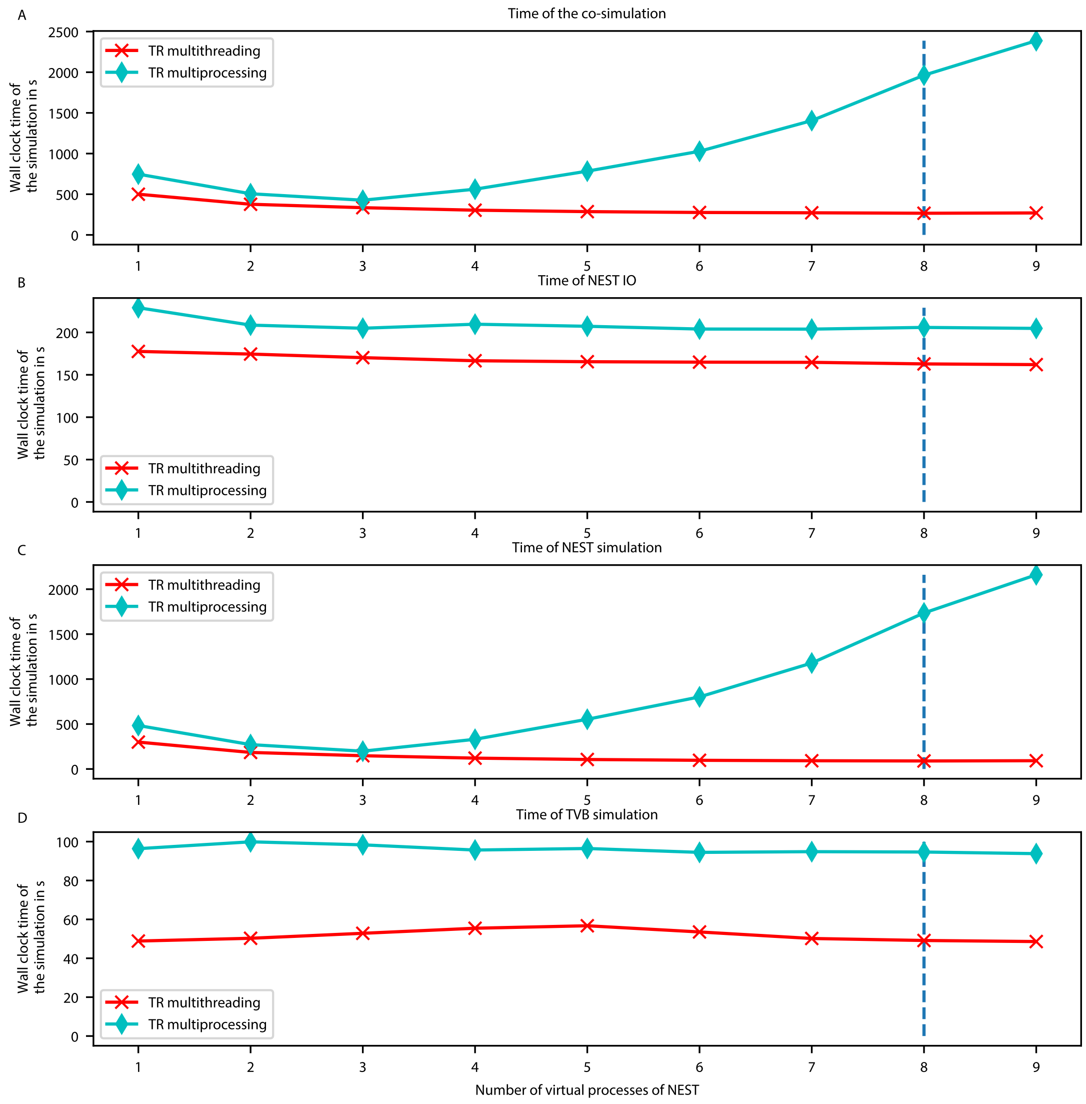

Figure 20. Compare the performance depending on parallelization strategies of the transformer modules
Performance is obtained for 1 second of simulated time on a computer (see Materials and Methods for more details). The reference implementation uses 1 MPI process, 2.0 ms to synchronize time between simulators for the simulation of 20000 neurons. Simulation time depends on the number of virtual processes used by NEST and the parallelization strategies of the transformer module (multiprocessing or multithreading). The cyan and red curves are associated with different parallelization strategies of the transfer module, respectively multiprocessing and multithreading. The horizontal blue line represents the number of cores of the computer **a** The total time of the co-simulation. **b** The ”IO” time of NEST **c** The ”simulation” time of NEST **d** The ”simulation” time of TVB

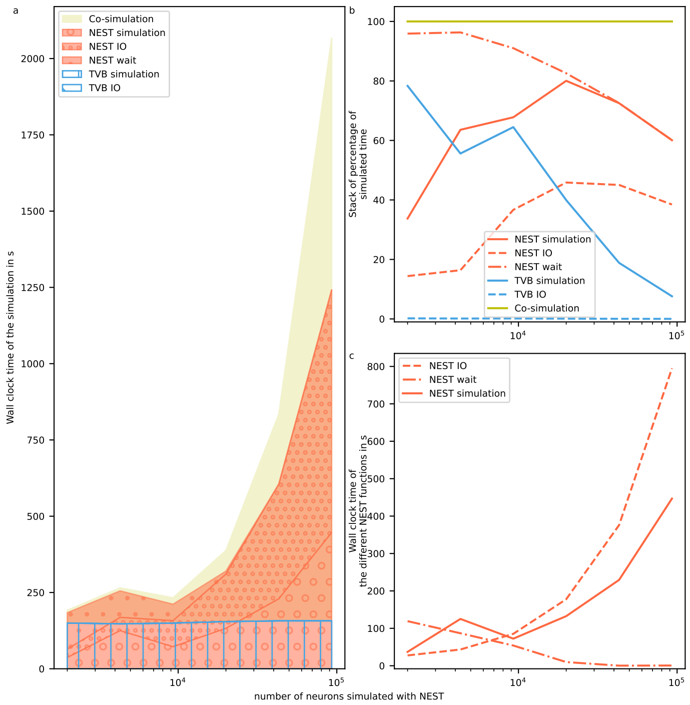

Figure 21. Performance of the co-simulation on a supercomputer for different number of neurons
Performance is obtained for 1 second of simulated time on a computer. The reference implementation uses 1 MPI process, 6 virtual processes/threads, 2.0 ms to synchronize time between simulators for the simulation of 20000 neurons. The node of Jusuf, the supercomputer, content 2 AMD EPYC 7742 @ 2.25 GHz * 64 cores * 2 threads, 256 (16x16) GB DDR4 with 3200 MHz, connected by InfiniBand HDR100 (Connect-X6). The transfer modules and TVB are on one node, and NEST is on one or multiple other nodes. Simulation time depends on the number of neurons simulated with NEST. **a** The wall clock time of the simulator depends on the number of neurons. The total time of the co-simulation is represented in yellow. The ”simulation”, ”IO” and ”wait” times of NEST are represented in the red surface with respectively hatches of big circles, small circles and points. The ”simulation” and ”IO” times of TVB are represented in the blue surface with respective hatches of horizontal lines and oblique lines. The ”simulation” time for TVB is constant. The sum of ”simulation” and ”IO” time of NEST is higher than the TVB ”simulation”. **b** The wall clock time for the co-simulation (yellow curve), NEST (red curves) and TVB (blue curves) by the total wall clock time. The solid, dashed and dashed-dotted curves are associated with ”simulation”, ”IO” and ”wait” time of NEST. The solid and dashed line is associated with ”simulation” and ”IO” time of TVB. The initialisation and configuration time increase with the number of neurons. **c** The contribution of NEST module to the total amount of the wall clock time normalizes between 0 and 100. Each contribution is reported as red curves in function of number of neurons. The solid, dashed and dashed-dotted curves represent ”simulation”, ”IO” and ”wait” time of NEST, respectively. The ”IO” time of NEST increases exponentially with the number of neurons and is higher than the ”simulation time when the number of neurons is higher than 6*1e4 of neurons.

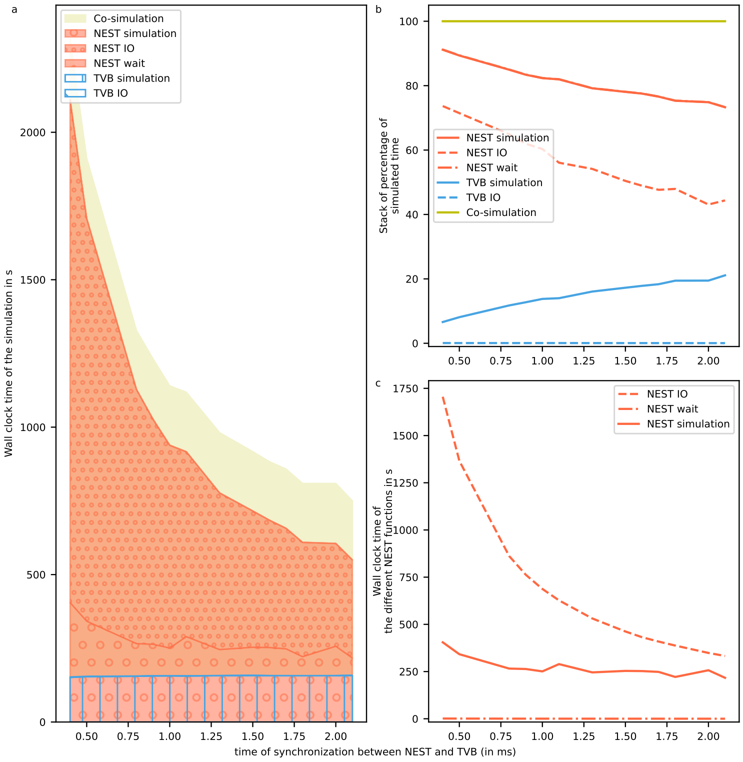

Figure 22. Performance of the co-simulation on a supercomputer for different time of synchronization between simulator
Performance is obtained for 1 second of simulated time on a computer. The reference implementation uses 1 MPI process, 6 virtual processes/threads, 2.0 ms to synchronize time between simulators for the simulation of 20000 neurons. The node of Jusuf, the supercomputer, content 2 AMD EPYC 7742 @ 2.25 GHz * 64 cores * 2 threads, 256 (16x16) GB DDR4 with 3200 MHz, connected by InfiniBand HDR100 (Connect-X6). The transfer modules and TVB are on one node, and NEST is on one or multiple other nodes. Simulation time depends on the synchronized time between simulators. **a** The wall clock time of the simulator. The simulation time reduces with the increase of the synchronization time between simulators. This reduction is due to the reduction of NEST ”IO” time. The total time of the co-simulation is represented in yellow. The ”simulation”, ”IO” and ”wait” times of NEST are represented in the red surface with respectively hatches of big circles, small circles and points. The ”simulation” and ”IO” times of TVB are represented in the blue surface with respective hatches of horizontal lines and oblique lines. The ”simulation” time for TVB is constant. The sum of ”simulation” and ”IO” time of NEST is higher than the TVB ”simulation”. **b** The wall clock time for different co-simulation modules normalized by the total wall clock time. All the curves are shown for an increase in the synchronized time between simulators. **c** The contribution of NEST module to the total amount of the wall clock time normalizes between 0 and 100. Each contribution is reported as red curves in function of synchronized time. The reduction follows a logarithm function.

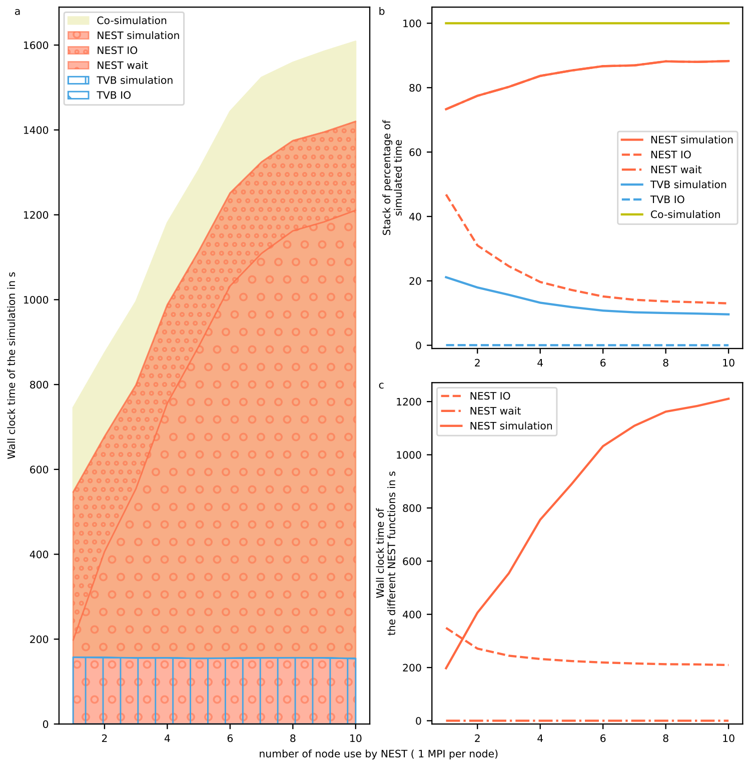

Figure 23. Performance of the co-simulation on a supercomputer for different number of node for NEST
Performance is obtained for 1 second of simulated time on a computer. The reference implementation use 1 MPI process, 6 virtual processes/threads, 2.0 ms to synchronize time between simulator for the simulation of 20000 neurons. The node of Jusuf, the supercomputer, content 2 AMD EPYC 7742 @ 2.25 GHz * 64 cores * 2 threads, 256 (16x16) GB DDR4 with 3200 MHz, connected by InfiniBand HDR100 (Connect-X6). The transfer modules and TVB are on one node, and NEST is on one or multiple other nodes. Simulation depends on the number of nodes used by NEST. **a** The wall clock time of the simulator as a function of the number of nodes used by NEST. The increase of the nodes creates overhead communication in side NEST because the network is small. Moreover, the minimum delay in the network is the same as the integration step, which creates an overhead of communication in NEST simulation. The wall clock time of the simulator depends on the number of neurons. The total time of the co-simulation is represented in yellow. The ”simulation”, ”IO” and ”wait” times of NEST are represented in the red surface with respectively hatches of big circles, small circles and points. The ”simulation” and ”IO” times of TVB are represented in the blue surface with respective hatches of horizontal lines and oblique lines. The ”simulation” time for TVB is constant. The sum of ”simulation” and ”IO” time of NEST is higher than the TVB ”simulation”. **b** The wall clock time for different co-simulation modules normalized by the total wall clock time. **c** The contribution of NEST module to the total amount of the wall clock time normalized between 0 and 100. The NEST ”IO” time remains constant with the increase in number of nodes.

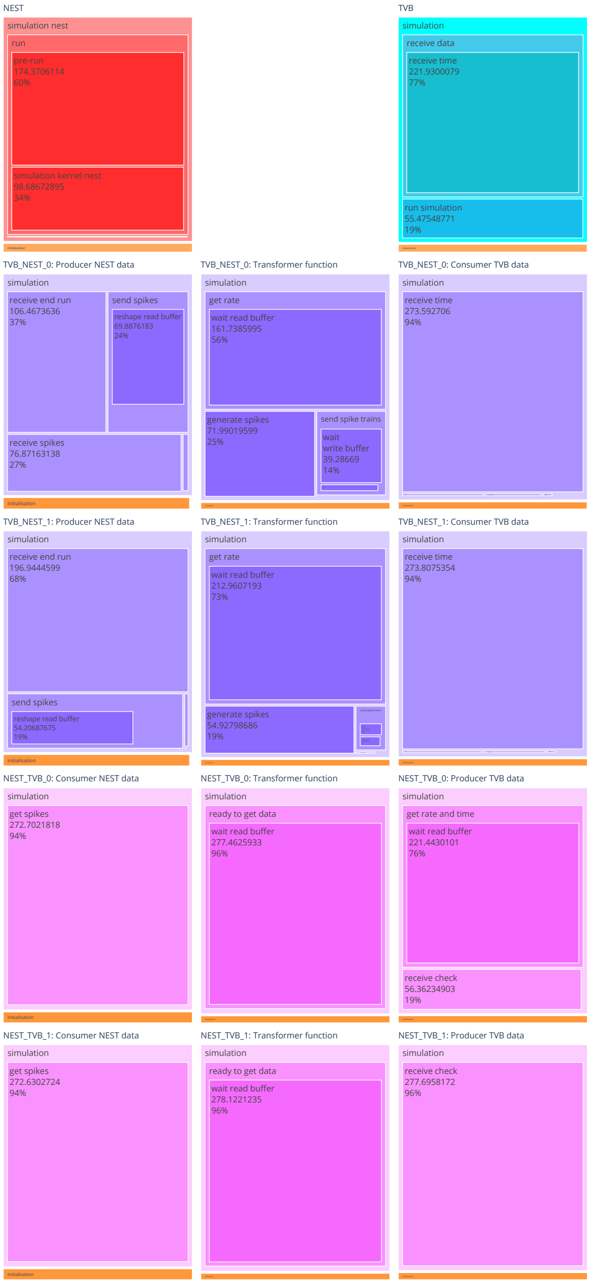

Figure 24. Details of the timer of one run for the reference configuration
This tree-map represents the timer for each component of the transfer module and for the modules NEST and TVB at the top. The orange bar under each box represents the time required for the initialisation. Each rectangle represents the time spent on each specific piece of code.

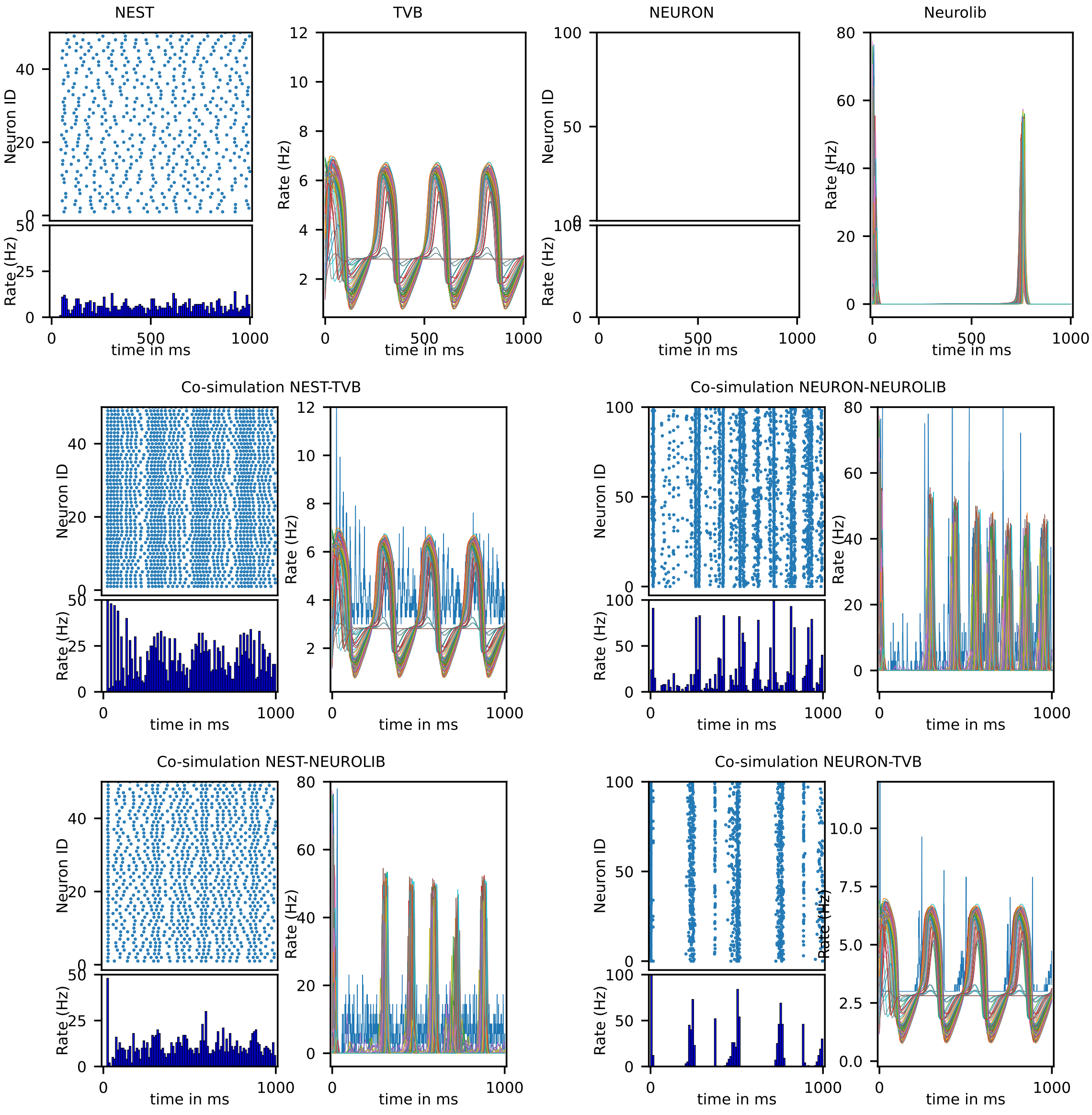

Figure 25. Proof of concept of replacing NEST and TVB with other simulators
For spiking neuron simulators (NEST and NEURON), the simulation output is the spike train of neurons (top graphic) and the firing rate of the population (bottom graphic: histogram of spike count with a bin of 10 ms). For neural mass simulators (TVB and Neurolib), the simulation output is the mean firing rate of the excitatory population.
The first row shows different examples without co-simulation on other simulators. One remark about the network used in NEURON, this network without external stimulation doesn't have any activities. The second and third rows display the result of the coupling example together simulated using co-simulation. The co-simulation results show the interaction of examples between them and the possibility of simulating these four different multiscale examples. The code is available here: https://github.com/multiscale-cosim/TVB-NEST-demo/tree/proof-concept

SUPPLEMENTARY REFERENCE

1 .[Dataset] Hahne J, Diaz S, Patronis A, Schenck W, Peyser A, Graber S, et al. NEST 3.0 (2021). doi:10.5281/zenodo.4739103.

2 .Sanz-Leon P, Knock SA, Spiegler A, Jirsa VK. Mathematical framework for large-scale brain network modeling in the virtual brain. *NeuroImage* **111** (2015) 385–430. doi:10.1016/j.neuroimage.2015.01.002.

3 .[Dataset] Gomes C, Thule C, Broman D, Larsen PG, Vangheluwe H. Co-simulation: A survey (2018).

4 .Chopard B, Borgdorff J, Hoekstra AG. A framework for multi-scale modelling. *Philosophical Transactions of the Royal Society A: Mathematical, Physical and Engineering Sciences* **372** (2014) 20130378. doi:10.1098/rsta.2013.0378. Publisher: Royal Society.

5 .Taveres-Cachat E, Favoino F, Loonen R, Goia F. Ten questions concerning co-simulation for performance prediction of advanced building envelopes. *Building and Environment* **191** (2021) 107570. doi:10.1016/j.buildenv.2020.107570.

6 .Sanz Leon P, Knock SA, Woodman MM, Domide L, Mersmann J, McIntosh AR, et al. The virtual brain: a simulator of primate brain network dynamics. *Front. Neuroinform.* **7** (2013) 10. doi:10.3389/fninf.2013.00010.

7 .Brette R, Gerstner W. Adaptive exponential integrate-and-fire model as an effective description of neuronal activity. *Journal of Neurophysiology* **94** (2005) 3637–3642. doi:10.1152/jn.00686.2005.

8 .Hagen E, Dahmen D, Stavrinou ML, Lindén H, Tetzlaff T, Albada V, et al. Hybrid scheme for modeling local field potentials from point-neuron networks. *Cereb. Cortex* **26** (2016) 4461–4496. doi:10.1093/cercor/bhw237.

9 .Shuman T, Aharoni D, Cai DJ, Lee CR, Chavlis S, Page-Harley L, et al. Breakdown of spatial coding and interneuron synchronization in epileptic mice. *Nat. Neurosci.* **23** (2020) 229–238. doi:10.1038/s41593-019-0559-0.

10 .Melozzi F, Woodman MM, Jirsa VK, Bernard C. The virtual mouse brain: A computational neuroinformatics platform to study whole mouse brain dynamics. *eNeuro* **4** (2017) ENEURO.0111–17.2017. doi:10.1523/ENEURO.0111-17.2017.

11 .Oh SW, Harris JA, Ng L, Winslow B, Cain N, Mihalas S, et al. A mesoscale connectome of the mouse brain. *Nature* **508** (2014) 207–214. doi:10.1038/nature13186.

12 .di Volo M, Romagnoni A, Capone C, Destexhe A. Biologically realistic mean-field models of conductance-based networks of spiking neurons with adaptation. *Neural Computation* **31** (2019) 653–680. doi:10.1162/neco_a_01173.

13 .Kuhn A, Aertsen A, Rotter S. Higher-order statistics of input ensembles and the response of simple model neurons. *Neural Computation* **15** (2003) 67–101. doi:10.1162/089976603321043702.
